# Explainable Brain Age Prediction using coVariance Neural Networks

**Published:** 2023-09-26

**Authors:** Saurabh Sihag, Gonzalo Mateos, Corey T. McMillan, Alejandro Ribeiro

**Affiliations:** University of Pennsylvania; University of Rochester; University of Pennsylvania; University of Pennsylvania

## Abstract

In computational neuroscience, there has been an increased interest in developing machine learning algorithms that leverage brain imaging data to provide estimates of “brain age” for an individual. Importantly, the discordance between brain age and chronological age (referred to as “brain age gap”) can capture accelerated aging due to adverse health conditions and therefore, can reflect increased vulnerability towards neurological disease or cognitive impairments. However, widespread adoption of brain age for clinical decision support has been hindered due to lack of transparency and methodological justifications in most existing brain age prediction algorithms. In this paper, we leverage coVariance neural networks (VNN) to propose an anatomically interpretable framework for brain age prediction using cortical thickness features. Specifically, our brain age prediction framework extends beyond the coarse metric of brain age gap in Alzheimer’s disease (AD) and we make two important observations: (i) VNNs can assign anatomical interpretability to elevated brain age gap in AD by identifying contributing brain regions, (ii) the interpretability offered by VNNs is contingent on their ability to exploit specific eigenvectors of the anatomical covariance matrix. Together, these observations facilitate an explainable perspective to the task of brain age prediction.

## 1 Introduction

Aging is characterized by progressive changes in the anatomy and function of the brain [1] that can be captured by different modalities of neuroimaging [2, 3]. Importantly, individuals can age at variable rates, a phenomenon described as “biological aging” [4]. Numerous existing studies based on a large spectrum of machine learning approaches study brain-predicted biological age, also referred to as brain age, which is derived from neuroimaging data [5–12]. Accelerated aging, i.e., when biological age is elevated as compared to chronological age (time since birth), may predict age-related vulnerabilities like risk for cognitive decline or neurological conditions like Alzheimer’s disease (AD) [13, 14]. In this domain, the metric of interest is *brain age gap*, i.e., the difference between brain age and chronological age. We use the notation Δ-Age to refer to the brain age gap.

Inferring Δ-age from neuroimaging data presents a unique statistical challenge as it is essentially a qualitative metric with no ground truth and is expected to be elevated in individuals with underlying neurodegenerative condition as compared to the healthy population[12, 15]. The existing machine learning approaches often rely on models that have been trained to predict chronological age for a healthy population and then applied to a cohort with neurodegenerative condition. A layman overview of the procedure of inferring Δ-Age is included in Appendix B. Several criticisms for brain age evaluation approaches have been identified that stem from the lack of transparency in the machine learning models. Major criticisms include the coarseness of Δ-Age that results in lack of specificity of brain regions contributing to the elevated brain age; and an unexplained reliance on the prediction accuracy for chronological age in the design of these machine learning models [5]. A significant portion of existing literature that studies brain age using deep learning models considers the ability of their models to accurately predict chronological age for healthy controls [16–18] as a relevant metric for assessing the quality of their methodological approach. Simultaneously, deep learning models that have a relatively moderate fit on the chronological age of healthy controls can also provide better insights into brain age than the ones with a tighter fit [17, 19]. Thus, there is a lack of conceptual clarity in the role of training to predict chronological age of healthy controls in predicting a meaningful Δ-age [20].

In this paper, we propose a principled framework for brain age prediction using cortical thickness data that accommodates interpretability by isolating the contributing brain regions to elevated Δ-Age in neurodegeneration. Our framework is based on the recently studied coVariance neural networks (VNNs) [21]. VNN is a graph convolutional network (GCN) with sample covariance matrix as the graph. The foundational analyses of VNNs in [21] showed that VNNs draw similarities with principal component analysis (PCA) while overcoming its significant drawbacks concerning reproducibility. Cortical thickness evolves with normal aging [22] and is impacted due to neurodegeneration [23, 24]. Thus, the age-related and disease severity related variations also appear in anatomical covariance matrices evaluated from the correlation among the cortical thickness measures across a population [25]. We focus our analysis on open access OASIS-3 dataset consisting of cortical thickness features from cognitively normal individuals and subjects in various stages of cognitive decline [26]. The utility of VNNs in predicting Δ-Age has been explored previously in [27] but no insights were provided regarding their explainability. See Appendix A for other relevant studies.

### Contributions.

Our contributions in this paper are summarized as follows.

- **VNNs provide anatomically interpretable Δ-Age:** Δ-Age in individuals with AD diagnosis was elevated as compared to healthy controls and significantly correlated with a clinical marker of dementia severity. Moreover, we could identify contributing brain regions to elevated Δ-Age by analyses of the outputs at the final layer of the VNNs. Hence, by exploiting the VNN architecture, we could characterize Δ-Age with anatomical interpretability (Fig. 2).
- **Anatomical interpretability correlate with eigenvectors of the anatomical covariance matrix:** Our experiments demonstrated that certain eigenvectors of the anatomical covariance matrix were strongly correlated with the features that facilitated anatomical interpretability for Δ-Age (Fig. 3). Thus, Δ-Age was linked to the ability of VNNs to exploit specific eigenvectors of the anatomical covariance matrix.

The aforementioned findings also helped clarify the role of the preliminary step of training the VNNs to predict chronological age of a healthy population in Δ-Age prediction. Specifically, this step equipped the VNNs with the ability to exploit the eigenvectors of the anatomical covariance matrix associated with elevated Δ-Age or accelerated aging in AD-driven neurodegeneration.

## 2 coVariance Neural Networks

We begin with a brief introduction to VNNs. VNNs inherit the architecture of GCNs and operate on sample covariance matrix as the graph [21]. A dataset consisting of n random, independent and identically distributed (i.i.d) samples, given by ${\text{x}}_{i}\in R^{m\times 1},\forall i\in \{1,\ldots ,n\}$, can be represented in matrix form as $X_{n}=\left[x_{1},\ldots ,x_{n}\right]$. Using $X_{n}$, the sample covariance matrix is estimated as

(1) $$C≜\frac{1}{n-1}\overset{n}{\underset{i=1}{\sum }}\left(x_{i}-\bar{x}\right){\left(x_{i}-\bar{x}\right)}^{⊤},$$

where $\overset{-}{\text{x}}$ is the sample mean of samples in $X_{n}$. The covariance matrix C can be viewed as the adjacency matrix of a graph representing the stochastic structure of the dataset $X_{n}$, where the m dimensions of the data can be thought of as the nodes of an m-node, undirected graph and its edges represent the pairwise covariances between different dimensions.

### 2.1 Architecture

Similar to GCNs that rely on convolution operations modeled by *linear-shift-and-sum* operators [28, 29], the convolution operation in a VNN is modeled by a coVariance filter, given by

(2) $$H\left(C\right)≜\overset{K}{\underset{k=0}{\sum }}h_{k}C^{k},$$

where scalar parameters ${\left\{h_{k}\right\}}_{k=0}^{K}$ are referred to as filter taps. The application of coVariance filter H(C) on an input x translates to combining information across different sized neighborhoods. For K>1, the convolution operation combines information across multi-hop neighborhoods (up to K-hop) according to the weights $h_{k}$ to form the output z=H(C)x.

A single layer of VNN is formed by passing the output of the coVariance filter through a nonlinear activation function σ(⋅) (e.g., ReLU, tanh) that satisfies $\sigma (u)=\left[\sigma \left(u_{1}\right),\ldots ,\sigma \left(u_{m}\right)\right]$ for $u=\left[u_{1},\ldots ,u_{m}\right]$. Hence, the output of a single layer VNN with input x is given by z=σ(H(C)x). The construction of a multi-layer VNN is formalized next.

**Remark 1** (Multi-layer VNN). *For an L-layer VNN, denote the coVariance filter in layer* ℓ *of the VNN by* $H_{\ell }(C)$ *and its corresponding set of filter taps by* ${ℋ}_{\ell }$. *Given a pointwise nonlinear activation function* σ(⋅), *the relationship between the input* ${\text{x}}_{\ell -1}$ *and the output* ${\text{x}}_{\ell }$ *for the* ℓ-*th layer is*

(3) $$x_{\ell }=\sigma \left(H_{\ell }\left(C\right)x_{\ell -1}\right)\text{ for }\ell \in \left\{1,\ldots ,L\right\},$$

*where* ${\text{x}}_{0}$ *is the input* x.

Furthermore, similar to other deep learning models, sufficient expressive power can be facilitated in the VNN architecture by incorporating multiple input multiple output (MIMO) processing at every layer. Formally, consider a VNN layer ℓ that can process $F_{\ell -1}$ number of m-dimensional inputs and outputs $F_{\ell }$ number of m-dimensional outputs via $F_{\ell -1}\times F_{\ell }$ number of filter banks [30]. In this scenario, the input is specified as $X_{\text{in}}=\left[{\text{x}}_{\text{in}}[1],\ldots ,{\text{x}}_{\text{in}}\left[F_{\text{in}}\right]\right]$, and the output is specified as $X_{\text{out}}=\left[x_{\text{out}}[1],\ldots ,x_{\text{out}}\left[F_{\text{out}}\right]\right]$. The relationship between the f-th output $x_{\text{out}}[f]$ and the input $x_{\text{in}}$ is given by $x_{\text{out}}[f]=\sigma \left(\sum_{g=1}^{F_{\text{in}}}\;H_{fg}(C){\text{x}}_{\text{in}}[g]\right)$, where $H_{fg}(C)$ is the coVariance filter that processes ${\text{x}}_{\text{in}}[g]$. Without loss of generality, we assume that $F_{\ell }=F,\forall \ell \in \{1,\ldots ,L\}$. In this case, the set of all filter taps is given by $ℋ=\left\{{ℋ}_{fg}^{\ell }\right\},\forall f,g\in \{1,\ldots ,F\},\ell \in \{1,\ldots ,L\}$, where ${ℋ}_{fg}={\left\{h_{fg}^{\ell }[k]\right\}}_{k=0}^{K}$ and $h_{fg}^{\ell }[k]$ is the k-th filter tap for filter $H_{fg}(C)$. Thus, we can compactly represent a multi-layer VNN architecture capable of MIMO processing via the notation Φ(x;C,ℋ), where the set of filter taps ℋ captures the full span of its architecture. We also use the notation Φ(x;C,ℋ) to denote the output at the final layer of the VNN. Various aspects of the VNN architecture are illustrated in Fig. 6 in Appendix C The VNN final layer output Φ(x;C,ℋ) is succeeded by a readout function that maps it to the desired inference outcome.

### 2.2 Properties of VNNs

The foundational analyses of VNNs in [21] established the following properties relevant to the data analysis in this paper.

#### VNNs and PCA.

Given the eigendecomposition of $C=V\Lambda V^{⊤}$, the spectral properties of the coVariance matrix are established by studying the projection of the coVariance filter output z=H(C)x on the eigenvectors V (similar to that for a graph filter using graph Fourier transform [31, 32]). Theorem 1 in [21] establishes the equivalence between processing data samples with PCA and processing data samples with a specific polynomial on the covariance matrix C. Hence, it can be concluded that input data is processed with VNNs, at least in part, by exploiting the eigenvectors of C.

#### Stability to perturbations in C.

VNNs are stable to perturbations in C [21, Theorem 3]. This property implies that the inference performance achieved by VNN is likely to be reproducible, and hence, robust to the number of samples n used to estimate the covariance matrix C.

#### VNNs are scale-free.

The filter taps in the coVariance filter in (2) are independent of the dimension of C. Hence, the covariance matrix C can readily be replaced with another covariance matrix $C^{\prime }$ (not necessarily of the same dimensionality as C) to process another dataset. By extension, the set of filter taps ℋ for a VNN is scale-free and can be used to process a dataset of arbitrary dimensionality. This observation is particularly relevant for data analysis in neuroimaging, where the number of features is highly variable across datasets, but different datasets contain similar information [33].

We leverage the aforementioned properties of VNNs in the context of brain age to demonstrate the relationships of the inference outcomes with the eigenvectors of C, robustness of the results to the number of samples used to estimate C, and cross-validate various findings by leveraging datasets of different dimensionalities.

### 2.3 VNN Learning

The VNN model is trained for a regression task using a dataset of m cortical thickness features and the chronological age for a population. Since the VNN architecture has F number of m-dimensional outputs in the final layer, Φ(x;C,ℋ) is of dimensionality m×F. The regression output is determined by a readout layer, which evaluates an unweighted mean of all outputs at the final layer of VNN. Therefore, the regression output for an individual with cortical thickness x is given by

(4) $$\hat{y}=\frac{1}{Fm}\overset{m}{\underset{j=1}{\sum }}\overset{F}{\underset{f=1}{\sum }}{\left[\Phi \left(x;C,ℋ\right)\right]}_{jf}.$$

Prediction using unweighted mean at the output implies that the VNN model exhibits permutation-invariance (i.e., the final output is independent of the permutation of the input features and covariance matrix) and VNN retains its scale-free property. Moreover, it allows us to associate a scalar output with each brain region among the m regions at the final layer. Specifically, we have

(5) $$p=\frac{1}{F}\overset{F}{\underset{f=1}{\sum }}{\left[\Phi \left(x;C,ℋ\right)\right]}_{f},$$

where p is the vector denoting the mean of filter outputs in the final layer’s filter bank. Note that the mean of all elements in p is the prediction $\overset{ˆ}{y}$ formed in (4). In the context of cortical thickness datasets, each element of p can be associated with a distinct brain region. Therefore, p is a vector of “regional contributions” to the output $\overset{ˆ}{y}$ by the VNN. This observation will be instrumental to establishing the interpretability offered by VNNs in the context of Δ-Age prediction in Section 3. For a regression task, the training dataset ${\left\{{\text{x}}_{i},y_{i}\right\}}_{i=1}^{n}$ (where ${\text{x}}_{i}$ are the cortical thickness data for an individual i with chronological age $y_{i}$) is leveraged to learn the filter taps in ℋ for the VNN Φ(⋅;C,ℋ) such that they minimize the training loss, i.e.,

(6) $${ℋ}_{\text{opt}}=\underset{ℋ}{\text{min}}\frac{1}{n}\overset{n}{\underset{i=1}{\sum }}\ell \left({\hat{y}}_{i},y_{i}\right),$$

where ${\overset{ˆ}{y}}_{i}$ is evaluated similarly to (4) and ℓ(⋅) is the mean-squared error (MSE) loss function.

## 3 Methods Overview for Brain Age Prediction

**Figure 1:**
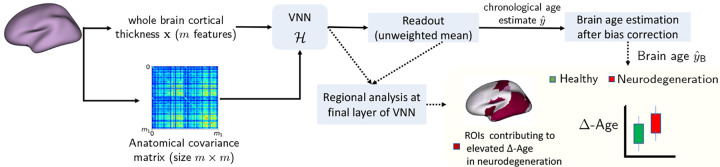
Workflow for brain age and Δ-Age prediction using VNNs.

In this section, we provide an overview of the brain age prediction framework based on VNNs (see Fig. 1 for a summary). Our results primarily focus on the dataset described below.

### OASIS-3 Dataset.

This dataset was derived from publicly available freesurfer estimates of cortical thickness (derived from MRI images collected by 3 Tesla scanners and hosted on central.xnat.org), as previously reported [26], and comprised of cognitively normal individuals (HC; n=611,age=68.38±7.62 years, 351 females) and individuals with AD dementia diagnosis and at various stages of cognitive decline (n=194,age=74.72±7.02 years, 94 females). The cortical thickness features were curated according to the Destrieux (DKT) atlas [34](consisting of m=148 cortical regions). For clarity of exposition of the brain age prediction method, any dementia staging to subdivide the group of individuals with AD dementia diagnosis into mild cognitive impairment (MCI) or AD was not performed, and we use the label AD+ to refer to this group. The individuals in AD+ group were significantly older than those in HC group (t-test: $p\text{-value}=2.46\times 10^{-23}$). The boxplots for the distributions of chronological age for HC and AD+ groups are included in Fig. 14 in Appendix H For 191 individuals in the AD+ group, the clinical dementia rating (CDR) sum of boxes scores evaluated within one year (365 days) from the MRI scan were available (CDR sum of boxes=3.45±1.74). CDR sum of boxes scores are commonly used in clinical settings to stage dementia severity [35] and were evaluated according to [36].

The findings obtained via the analyses of the OASIS-3 dataset were validated using a smaller, independent dataset (described in Appendix G).

### 3.1 Training the VNNs on HC group

We first train the VNN model to predict chronological age using the cortical thickness features for the HC group. This enables the VNN models to capture the information about healthy aging from the cortical thickness and associated anatomical covariance matrix. The hyperparameters for the VNN architecture and learning rate of the optimizer were chosen according to a hyperparameter search procedure using the package Optuna [37]. The VNN model had L=2-layers with a filter bank, such that we had F=5, and 6 filter taps in the first layer and 10 filter taps in the second layer. The learning rate for the Adam optimizer was set to 0.059. The number of learnable parameters for this VNN was 290. The HC group was split into a 90/10 training/test split, and the covariance matrix was set to be the anatomical covariance evaluated from the training set. A part of the training set was used as a validation set and the other used for training the VNN model. We trained a set of 100 VNNs on different permutations of the training data. The training process was similar for all VNNs and is described in Appendix D No further training was performed for the VNN models in the subsequent analyses.

### 3.2 Analyses of regional residuals in AD+ and HC groups

Next, the VNN models trained to predict the chronological age for the HC group and (5) were adopted to study the effect of neurodegeneration on brain regions for AD+ group. Since the impact of neurodegeneration is expected to appear in the anatomical covariance matrix, we report the results when anatomical covariance $C_{\text{HA}}$ from the combined cortical thickness data of HC and AD+ groups was deployed in the trained VNN models. Because of the stability property of VNNs [21, Theorem 3], the inference drawn from VNNs is expected to be stable to the composition of combined HC and AD+ groups used to estimate the anatomical covariance matrix $C_{\text{HA}}$.

For every individual in the combined dataset of HC and AD+ groups, we processed their cortical thickness data x through the model $\Phi \left(x;C_{\text{HA}},ℋ\right)$ where parameters ℋ were learned in the regression task on the data from HC group as described previously. Hence, the vector of mean of all final layer outputs for cortical thickness input x is given by $p=\frac{1}{F}\sum_{f=1}^{F}\;\left[\Phi {\left(x;C_{\text{HA}},ℋ\right]}_{f}\right.$ and the VNN output is $\overset{ˆ}{y}=\frac{1}{148}\sum_{j=1}^{148}\;[p{]}_{j}$. Furthermore, we define the residual for feature a (or brain region represented by feature a in this case) as

(7) $$[r{]}_{a}≜[p{]}_{a}-\hat{y}.$$

Thus, (7) allows us to characterize the residuals with respect to the VNN output $\overset{ˆ}{y}$ at the regional level, where brain regions are indexed by a. Henceforth, we refer to the residuals (7) as “regional residuals”. Recall that these are evaluated for an individual with cortical thickness data x.

In our experiments, for a given VNN model, the residual vector r was evaluated for every individual in the OASIS-3 dataset. Also, the population of residual vectors for the HC group is denoted by $r_{\text{HC}}$, and that for individuals in the AD+ group by $r_{\text{AD}+}$. The length of the residual vectors is the same as the number of cortical thickness features (i.e., m=148). Since each element of the residual vector is associated with a distinct brain region, ANOVA was used to test for group differences between individuals in HC and AD+ groups. Also, since elevation in Δ-Age is the biomarker of interest in this analysis, we hypothesized that the brain regions that exhibited higher means for regional residuals for AD+ group than HC group would be the most relevant to capturing accelerated aging. Hence, the results are reported only for brain regions that showed elevated regional residual distribution in AD+ group with respect to HC group. Further, the group difference between AD+ and HC groups in the residual vector element for a brain region was deemed significant if it met the following criteria: i) the corrected p-value (Bonferroni correction) for the clinical diagnosis label in the ANOVA model was smaller than 0.05; and ii) the uncorrected p-value for clinical diagnosis label in ANCOVA model with age and sex as covariates was smaller than 0.05. See Appendix F for an example of this analysis.

Recall that 100 distinct VNNs were trained as regression models on different permutations of the training set of cortical thickness features from HC group. We used these trained models to establish the robustness of observed group differences in the distributions of regional residuals.

#### Deriving regional profile for Δ-Age from the robustness of findings from regional analyses.

We performed the regional analysis described above corresponding to each trained VNN model and tabulated the number of VNN models for which a brain region was deemed to be associated with a significantly elevated regional residual for the AD+ group. A higher number of VNN models isolating a brain region as significant suggested that this region was likely to be a highly robust contributor to accelerated aging in the AD+ group.

### 3.3 Individual-level Brain Age Prediction

Finally, a scalar estimate for the brain age was obtained from the VNN regression output through a procedure consistent with the existing studies in this domain. Note that 100 VNNs provide 100 estimates $\overset{ˆ}{y}$ of the chronological age for each subject. For simplicity, we consider $\overset{ˆ}{y}$ to be the mean of these estimates. A systemic bias in the gap between $\overset{ˆ}{y}$ and y may potentially exist when the correlation between $\overset{ˆ}{y}$ and y is smaller than 1. Such a bias can confound the interpretations of brain age due to underestimation for older individuals and overestimation for younger individuals [38]. Therefore, to correct for this age-induced bias in $\overset{ˆ}{y}-y$, we adopted a linear regression model-based approach [38, 39]. Specifically, the following bias correction steps were applied on the VNN estimated age $\overset{ˆ}{y}$ to obtain the brain age ${\overset{ˆ}{y}}_{\text{B}}$ for an individual with chronological age y:

**Step 1**. Fit a regression model for the HC group to determine scalars α and β in the following model:

(8) $$\hat{y}-y=\alpha y+\beta \text{.}$$

**Step 2**. Evaluate brain age ${\overset{ˆ}{y}}_{\text{B}}$ as follows:

(9) $${\hat{y}}_{\text{B}}=\hat{y}-\left(\alpha y+\beta \right).$$

The gap between ${\overset{ˆ}{y}}_{\text{B}}$ and y is the Δ-Age and is defined below. For an individual with cortical thickness x and chronological age y, the brain age gap Δ-Age is formally defined as

(10) $$\Delta \text{-Age}≜{\hat{y}}_{\text{B}}-y,$$

where ${\overset{ˆ}{y}}_{\text{B}}$ is determined from the VNN age estimate $\overset{ˆ}{y}$ and y according to steps in (8) and (9). The age-bias correction in (8) and (9) was performed only for the HC group to account for bias in the VNN estimates due to healthy aging, and then applied to the AD+ group. Further, the distributions of Δ-Age were obtained for all individuals in HC and AD+ groups.

Δ-Age for AD+ group was expected to be elevated as compared to HC group as a consequence of elevated regional residuals derived from the VNN model. To elucidate this, consider a toy example with two individuals of the same chronological age y, with one belonging to the AD+ group and another to the HC group. Equation (9) suggests that their corresponding VNN outputs (denoted by ${\overset{ˆ}{y}}_{\text{AD}+}$ for individual in the AD+ group and ${\overset{ˆ}{y}}_{\text{HC}}$ for individual in the HC group) are corrected for age-bias using the same term αy+β. Hence, Δ-Age for the individual in the AD+ group will be elevated with respect to that from the HC group only if the VNN prediction ${\overset{ˆ}{y}}_{\text{AD}+}$ is elevated with respect to ${\overset{ˆ}{y}}_{\text{HC}}$. Since the VNN predictions ${\overset{ˆ}{y}}_{\text{AD}+}$ and ${\overset{ˆ}{y}}_{\text{HC}}$ are proportional to the unweighted aggregations of the estimates at the regional level [see (4) and (5)], larger ${\overset{ˆ}{y}}_{\text{AD}+}$ with respect to ${\overset{ˆ}{y}}_{\text{HC}}$ can be a direct consequence of a subset of regional residuals [see (7)] being robustly elevated in AD+ group with respect to HC group across the 100VNN models. When the individuals in this example have different chronological age, the age-bias correction is expected to remove any variance due to chronological age in Δ-Age. We also verified that the differences in Δ-Age for AD+ and HC group were not driven by age or sex differences via ANCOVA with age and sex as covariates.

## 4 Results

### 4.1 Chronological age prediction for the HC group

The performance achieved by the VNNs on the chronological age prediction task for the HC group has been summarized over the 100 nominal VNN models. VNNs achieved a mean absolute error (MAE) of 5.82 ± 0.13 years and Pearson’s correlation of 0.51 ± 0.078 between the chronological age estimates and the ground truth on the test set. Moreover, on the complete dataset, the MAE was 5.44 ± 0.18 years and Pearson’s correlation was 0.47 ± 0.074. Thus, the trained VNNs were not overfit on the training set.

Next, for every individual in the HC group, we evaluated the mean of the inner products (also equivalently referred to as dot product) between the vectors of contributions of every brain region [p in (5)] and the eigenvectors of the anatomical covariance matrix for all 100VNN models. The strongest alignment was present between the first eigenvector of the anatomical covariance matrix (i.e., the eigenvector associated with the largest eigenvalue) and the vectors of regional contributions to the VNN output (0.987±0.0005 across the HC group), with relatively smaller associations for second (0.051±0.003), third (0.075±0.004), and fourth (0.094±0.003) eigenvectors. Additional details are included in Appendix E. Thus, the VNNs exploited the eigenvectors of the anatomical covariance matrix to achieve the learning performance in this task. The first eigenvector of the anatomical covariance matrix predominantly included bilateral anatomic brain regions in the parahippocampal gyrus, precuneus, inferior medial temporal gyrus, and precentral gyrus. These findings were cross-validated on the HC group by the VNNs that had been trained on a different dataset (Appendix G).

We remark that several existing studies on brain age prediction have utilized deep learning and other approaches to report better MAE on their respective healthy populations [16–18, 40]. In contrast, our contribution here is conceptual, where we have explored the properties of VNNs when they are trained to predict the chronological age for the HC group. Subsequently, our primary focus in the context of brain age is on demonstrating the anatomical interpretability offered by VNNs and relevance of eigenvectors of the anatomical covariance matrix. Thus, we further provide the insights that have not been explored (or are infeasible to obtain) for most existing brain age evaluation frameworks based on less transparent deep learning models.

### 4.2 Analyses of regional residuals derived from VNNs reveal regions characteristic of AD

**Figure 2:**
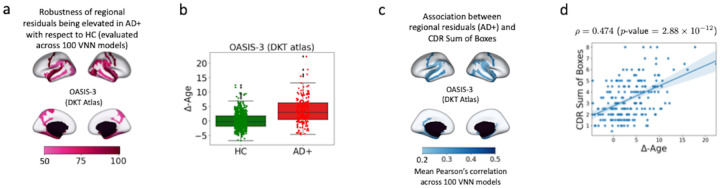
Interpretable Δ-Age evaluation in OASIS-3 dataset. Panel **a** displays the robustness of the significantly elevated regional residuals for AD+ group with respect to HC group for different brain regions. For every VNN model in the set of 100 nominal VNN models that were trained on HC group, the analyses of regional residuals helped isolate brain regions that corresponded to significantly elevated regional residuals in AD+ group with respect to HC group. After performing this experiment for 100VNN models, the robustness of the observed significant effects in a brain region was evaluated by calculating the number of times a brain region was identified to have significantly elevated regional residuals in AD+ group with respect to HC group. The number of times a brain region was linked with significantly elevated regional residuals in AD+ group with respect to HC group is projected on the brain template. Panel **b** displays the distribution of Δ-Age for HC and AD+ groups. The elevated brain age effect here is characterized by regional profile in Panel a. Panel **c** projects the mean Pearson’s correlation between regional residuals (derived for each VNN model in the set of 100 nominal VNN models) and CDR sum of boxes for AD+ group on the brain template. Panel **d** displays the scatter plot for CDR sum of boxes versus Δ-Age in AD+ group. The correlation between Δ-Age and CDR sum of boxes could be attributed to the observations made in Panel **c**.

Figure 2a displays the robustness (determined via analyses of 100VNN models) for various brain regions being associated with significantly larger residual elements for the AD+ group than the HC group. The most significant regions with elevated regional residuals in AD+ with respect to HC were concentrated in bilateral inferior parietal, temporal, entorhinal, parahippocampal, subcallosal, and precentral regions. All these brain regions, except for precentral and subcallosal, mirrored the cortical atrophy (Fig. 15. in supplementary material), and these regions are known to be highly interconnected with hippocampus [41]. Hence, brain regions characteristic of AD had significant differences in regional residual distributions for AD+ group as compared to HC group. Similar findings were recovered by the analysis of the regional residuals derived from the VNNs that were trained on an independent dataset (Appendix G).

Although the results in Fig. 2a provided a meaningful regional profile for AD+ group, we further performed exploratory analysis to check whether the regional residuals had any clinical significance. To this end, we evaluated the Pearson’s correlations between CDR sum of boxes and the regional residuals derived from final layer VNN outputs for the AD+ group for all 100VNN models. Interestingly, the brain regions with the largest correlations with the CDR sum of boxes scores in Fig. 2c were concentrated in the parahippocampal, medial temporal lobe, and temporal pole regions (also isolated in Fig. 2a). This observation provides the evidence that the regional residuals for the AD+ group that led to the result in Fig. 2a could predict dementia severity.

### 4.3 Δ-Age is elevated in AD+ group and correlated with CDR

We evaluated the Δ-Age for HC and AD+ groups according to the procedure specified in Section 3.3 We also investigated the Pearson’s correlation between Δ-Age and CDR sum of boxes scores in AD+ group. Figure 2b illustrates the distributions for Δ-Age for HC and AD+ groups (Δ-Age for HC:0±2.83 years, Δ-Age for AD+:3.54±4.49 years). The difference in Δ-Age for AD+ and HC groups was significant (Cohen’s d=0.942, ANCOVA with age and sex as covariates: $p-\text{value}<10^{-20}$, partial $\left.\eta^{2}=0.158\right)$. Also, age and sex were not significant in ANCOVA (p-value>0.4 for both). Hence, the group difference in Δ-Age for the two groups was not driven by the difference in the distributions of their chronological age. Figure 2d plots CDR sum of boxes scores versus Δ-Age for the AD+ group. Pearson’s correlation between CDR sum of boxes score and Δ-Age was 0.474 $\left(p\right.-\text{value}\left.=2.88\times 10^{-12}\right)$, thus, implying that the Δ-Age evaluated for AD+ group captured information about dementia severity. Hence, as expected, the Δ-Age for AD+ was likely to be larger with an increase in CDR sum of boxes scores. For instance, the mean Δ-Age for individuals with CDR sum of boxes greater than 4 was 6.04 years, and for CDR sum of boxes ≤ 4 was 2.42 years.

Given that the age-bias correction procedure is a linear transformation of VNN outputs, it can readily be concluded that the statistical patterns for regional residuals in Fig. 2a and Fig. 2c lead to elevated Δ-Age and correlation between Δ-Age and CDR sum of boxes scores. Therefore, our framework provides a feasible way to characterize accelerated biological aging in AD+ group with a regional profile. Additional figures and details pertaining to VNN outputs and brain age before and after the age-bias correction was applied are included in Appendix H.

### 4.4 Regional residuals derived from VNNs trained on OASIS-3 are correlated with eigenvectors of the anatomical covariance matrix

**Figure 3:**
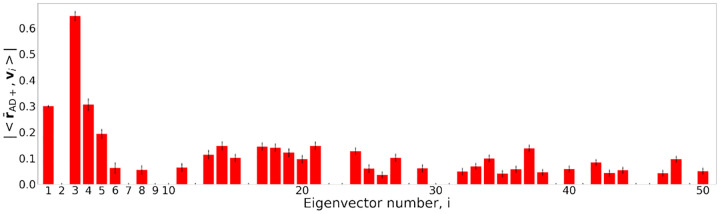
Bar plots of the mean inner products between the normalized vector of regional residuals (norm = 1) of VNN outputs (VNNs trained on OASIS-3) obtained from AD+ group and the eigenvectors of $C_{\text{HA}}$ (covariance matrix of combined HC and AD+ group) with respective standard deviations as error bars. Results with coefficient of variation > 30% across the AD+ group have been excluded.

**Figure 6:**
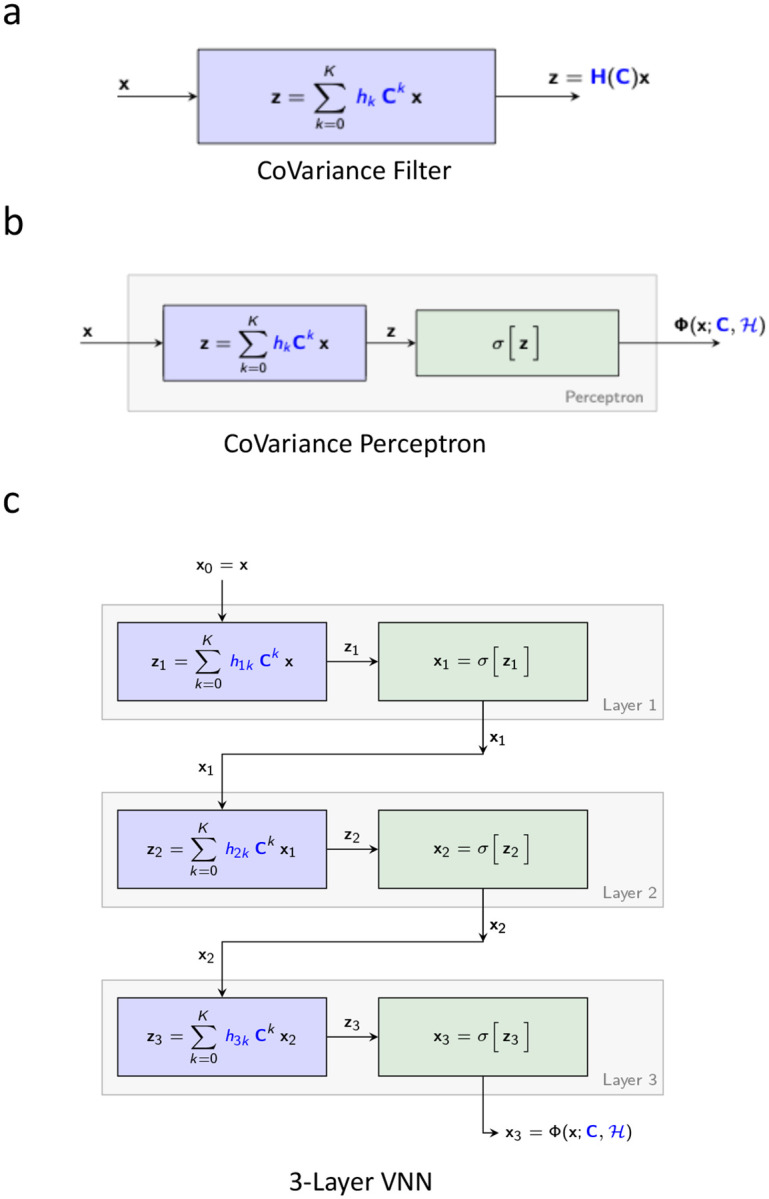
Basics of VNN architecture. Panel **a** illustrates that the coVariance filter **H**(**C**) is a polynomial in **C** and its application on input **x**. Panel **b** shows the construction of a coVariance perceptron based on coVariance filter **H**(**C**) and pointwise nonlinearity σ. coVariance perceptron specifies one layer of VNN. Panel **c** shows a basic multi-layer VNN architecture formed by stacking three coVariance perceptrons.

**Figure 14:**
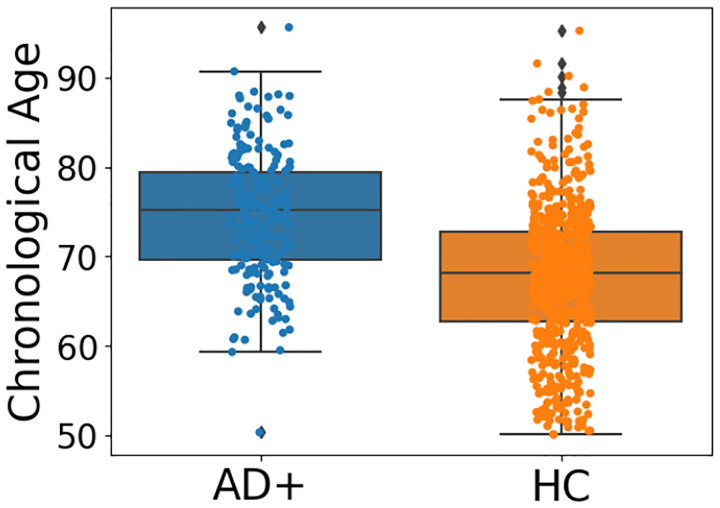
Distribution of chronological age in AD+ and HC groups.

**Figure 15:**
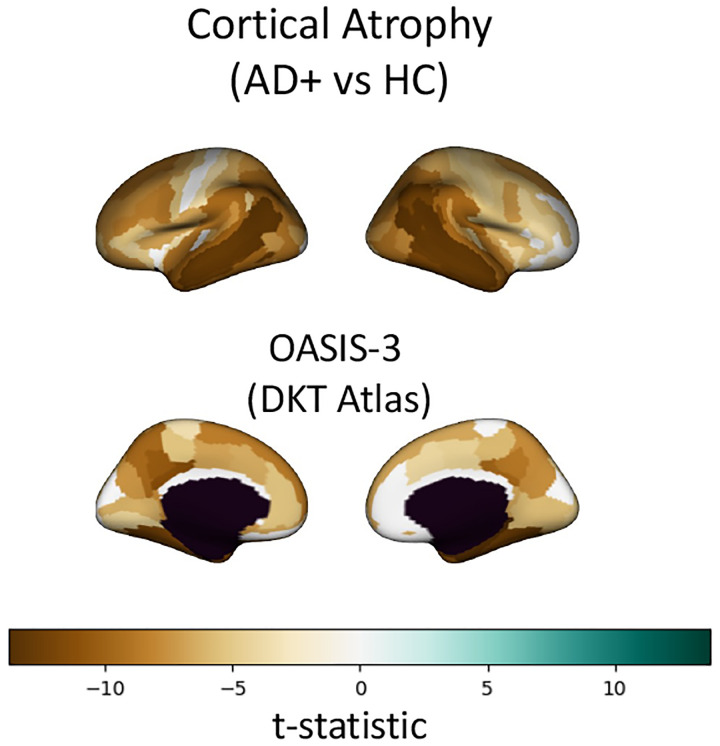
Results of group differences in cortical thickness between AD+ and HC groups. Regions with significant differences (two-sided t-test, Bonferroni corrected p-value < 0.05) are identified and the corresponding t-statistics are projected on a brain template. Negative t-statistic for a brain region suggests that the AD+ group had significant cortical atrophy in that region as compared to HC group.

We further investigated the relationship between regional residuals derived from VNNs and the eigenvectors of $C_{\text{HA}}$ to determine whether any specific eigenvectors (principal components) of $C_{\text{HA}}$ were instrumental to recover the findings in Fig. 2b. For this purpose, we evaluated the inner product of normalized residual vectors (norm = 1) obtained from VNNs and the eigenvectors of the covariance matrix $C_{\text{HA}}$ for the individuals in AD+ group. The normalized residual vector is denoted by ${\overset{-}{r}}_{\text{AD}+}$. For every individual, the mean of the absolute value of the inner product $\left|\left\langle {\overset{-}{r}}_{\text{AD}+},v_{i}\right\rangle \right|$ (where $v_{i}$ is the i-th eigenvector of $C_{\text{HA}}$) was evaluated for the 100VNN models that were used to derive the results in Fig. 2. Figure 3 plots the mean inner product for eigenvectors associated with 50 largest eigenvalues of $C_{\text{HA}}$. The three largest mean correlations with the regional residuals in AD+ group were observed for the third eigenvector of $C_{\text{HA}}\left(\left|<{\overset{-}{r}}_{\text{AD}+},v_{3}\right\rangle |=0.645\pm 0.016\right)$, fourth eigenvector $\left(\left|\left\langle {\overset{-}{r}}_{\text{AD}+},v_{4}\right\rangle \right|=0.305\pm 0.02\right)$, and the first eigenvector $\left(\left|\left\langle {\overset{-}{r}}_{\text{AD}+},v_{1}\right\rangle \right|=\right.0.299\pm 0.001)$. Similar correlation patterns between the regional residuals and the eigenvectors of $C_{HA}$ for the AD+ group were recovered by the VNNs that had been trained on an independent dataset and used to process OASIS-3 dataset (Appendix G). These eigenvectors are plotted on a brain template in the expanded Fig. 9b in the supplementary material. Inspection of the first, third, and fourth eigenvector of $C_{\text{HA}}$ suggested that subcallosal, entorhinal, parahippocampal and temporal pole regions could be the most relevant contributors to the elevated Δ-Age in AD+ group in Fig. 2

### 4.5 Additional Experiments

#### Cross-validation:

Using the VNNs trained to predict chronological age for an independent dataset of healthy population (cortical thickness curated according to a different brain atlas), we recovered a regional profile similar to Fig. 2a on the OASIS-3 dataset. Furthermore, the regional residuals derived from this set of VNNs had similar correlation patterns with the eigenvectors of $C_{\text{HA}}$. This observation and Fig. 3 led to the conclusion that training to predict chronological age of a healthy population was instrumental for the VNNs to gain the ability to exploit eigenvectors of the anatomical covariance matrix that are relevant to brain age prediction. See Appendix G for details.

#### Stability to perturbations in $C_{HA}$ :

As a consequence of the stability of VNNs, we observed that the regional profile for Δ-Age in Fig. 2a was stable even when the covariance matrix $C_{\text{HA}}$ was estimated by a variable composition of individuals from the HC and AD+ group (Appendix J).

#### Anatomical covariance matrix and brain age:

Use of anatomical covariance matrix derived from only HC group provides results consistent with Fig. 2, albeit with a slightly smaller group difference between the Δ-Age distributions for HC and AD+ groups. See Appendix K for details.

## 5 Discussion

Our study has provided a foundational contribution to the methodology of brain age prediction. Anatomical interpretability of Δ-Age was derived from the features extracted at the final layer of the VNNs and these features were correlated with marker of dementia severity and certain eigenvectors of the anatomical covariance matrix. Importantly, training the VNNs to predict chronological age helped fine tune their parameters to exploit the relevant eigenvectors of the anatomical covariance matrix. Thus, the role of the age-bias correction step was restricted to projecting the VNN outputs onto a space where one could infer accelerated biological aging with respect to the chronological age from a layman’s perspective. By associating Δ-Age with a regional profile, VNNs also provide a feasible tool to distinguish pathologies if the distributions of Δ-Age for them are overlapping. Moreover, a near-prefect chronological age prediction for healthy controls by itself may not be a determinant of the quality of brain age prediction in neurodegeneration. A larger focus is needed on principled statistical approaches for brain age prediction that can capture the factors that lead to accelerated aging. Locally interpretable and theoretically grounded deep learning models such as VNNs can provide a feasible, promising future direction to build statistically and conceptually legitimate brain age prediction models in broader contexts. Incorporating other modalities of neuroimaging or alternative metrics of aging other than chronological age (such as DNA methylation aging [42]) in the training of VNNs provide promising future directions that can help improve our understanding of aging.

### Limitations.

We provide findings derived on a single dataset (although corroborated using VNNs trained on an independent dataset) and further investigation on larger and more diverse datasets may improve the confidence in our conclusions. Existing studies, including this paper, fall short at concretely defining the notion of optimal brain age. From a broader perspective, quantifying biological age even for a healthy population is a complex task due to various factors that can contribute to accelerated aging in the absence of an adverse health condition [43–45]. Moreover, the impacts of the quality of MRI scans and brain atlases across datasets on Δ-Age must also be explored.

## Figures and Tables

**Figure 4:**
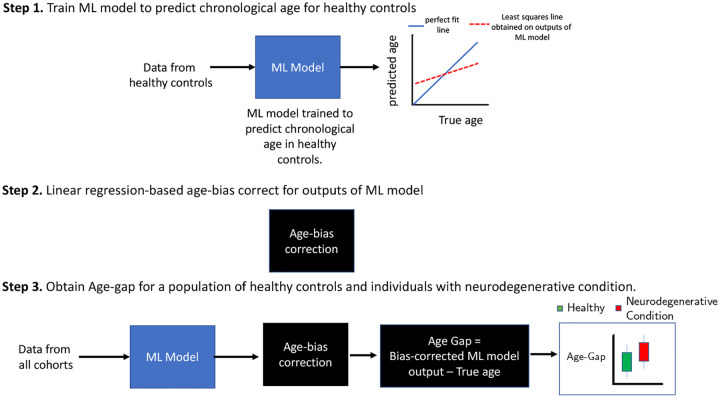
A general overview of brain age evaluation using machine learning algorithms in the existing literature. **Step 1** consists of training a machine learning (ML) model to predict chronological age (true age) for healthy controls. If the correlation between predicted age and true age is smaller than 1, an age-bias exists in ML model outputs as the age for older individuals tends to be under-estimated and that for younger individuals tends to be over-estimated. To correct for this bias, a linear regression based model is applied on the ML model outputs in **Step 2**. Under the hypothesis that ML model can capture accelerated aging in age-related neurodegeneration, it is expected that Δ-Age for individuals with neurodegeneration will be significantly higher than those of healthy controls **(Step 3)**.

**Figure 5:**
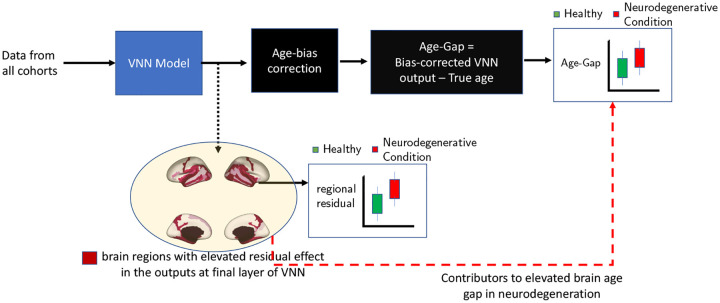
Interpretability offered by VNNs in brain age prediction. By analyzing the final layer outputs of VNNs, we can isolate brain regions that have larger regional residuals for individuals with AD with respect to healthy controls. Furthermore, the elevated regional residuals in these brain regions eventually contribute to elevated Δ-Age after age-bias correction.

**Figure 7:**
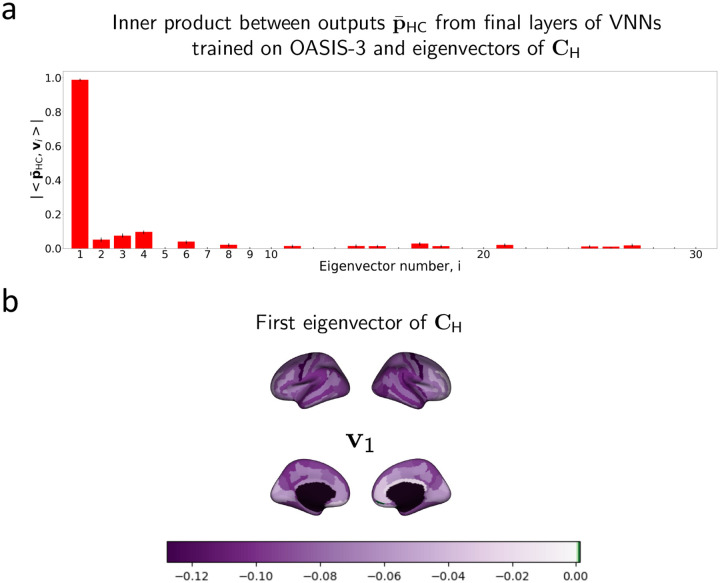
Inner product between the normalized vector of regional contributions to the VNN outputs $\left({\overset{-}{\text{p}}}_{\text{HC}}\right)$ and eigenvectors of ${\text{C}}_{\text{H}}$ (anatomical covariance matrix for HC group in OASIS-3). Panel **a** illustrates a bar plot for $\left|\left\langle {\overset{-}{p}}_{\text{HC}},v_{i}\right\rangle \right|$ for i∈{1,…,30}, where $v_{i}$ is the i-th eigenvector (principal component) of covariance matrix $C_{\text{H}}$ and associated with i-largest eigenvalue in terms of magnitude and the vectors of regional contributions, ${\overset{-}{p}}_{\text{HC}}$ were obtained by VNNs that were trained on OASIS-3 dataset. The inner product results for eigenvectors with coefficient of variation greater than 30% across the HC group of OASIS-3 were excluded (and hence, their respective entries set as 0). For every individual in HC group, the associations between their corresponding vector of regional contributions, ${\overset{-}{p}}_{\text{HC}}$ and eigenvectors of $C_{\text{H}}$ were evaluated over 100 nominal VNN models. The first eigenvector $\left(v_{1}\right)$ had the largest association. The eigenvector $v_{1}$ is plotted on a brain template in Panel **b**.

**Figure 8:**
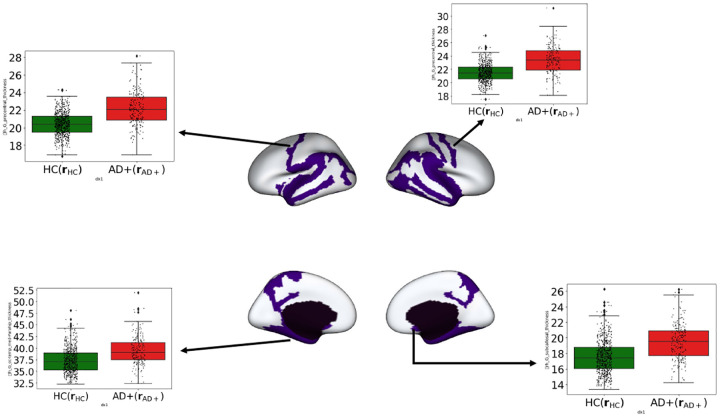
Results depicting the brain regions with significantly elevated regional residuals for AD+ group with respect to HC group in OASIS-3. The results here were derived by a VNN model that was trained as a regression model to predict chronological age from cortical thickness data for HC group in OASIS-3. Box plots depicting the distributions of regional residuals in the HC and AD+ groups are shown for a few representative regions.

**Figure 9:**
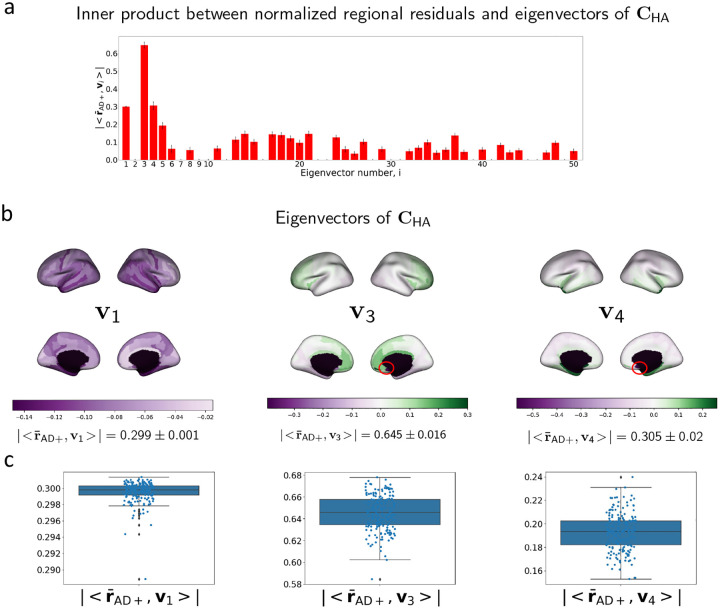
Mean inner product between the normalized vector of regional residuals (norm = 1) of VNN outputs (VNNs trained on OASIS-3) obtained from AD+ group and the eigenvectors of ${\text{C}}_{\text{HA}}$ (covariance matrix of combined HC and AD+ group), and eigenvector plots. Panel **a** illustrates a bar plot for $\left|\left\langle {\overset{-}{r}}_{\text{AD}+},v_{i}\right\rangle \right|$ for i∈{1,…,50}, where $v_{i}$ is the i-th eigenvector of covariance matrix $C_{\text{HA}}$ associated with its i-largest eigenvalue. The bars are evaluated from the mean of $\left|\left\langle {\overset{-}{r}}_{\text{AD}+},v_{i}\right\rangle \right|$ obtained for individuals in the AD+ group (results for eigenvectors associated with coefficient of variation of $\left|\left\langle {\overset{-}{r}}_{\text{AD}+},v_{i}\right\rangle \right|$ larger than 30% across the AD+ group excluded). For every individual in AD+ group, the associations of its regional residuals with eigenvectors of $C_{\text{HA}}$ were evaluated as the mean of those over 100 nominal VNN models (trained on the OASIS-3 dataset). The eigenvectors associated with top three largest values for $\left|\left\langle {\overset{-}{r}}_{\text{AD}+},v_{i}\right\rangle \right|$ are plotted on the brain template in Panel **b**. Subcallosal region in the right hemisphere was associated with the element with the largest magnitude in $v_{3}$ and $v_{4}$ and is highlighted with a red circle in the corresponding plots. Panel **c** displays the boxplots for the distributions of $\left|\left\langle {\overset{-}{r}}_{\text{AD}+},v_{i}\right\rangle \right|$ for $v_{1},v_{3}$, and $v_{4}$ across the AD+ group. Hence, the largest association was observed for the fourth eigenvector, which spanned the bilateral subcallosal, entorhinal, parahippocampal, and temporal pole regions. The third eigenvector spanned the subcallosal region in the right hemisphere. The first eigenvector spanned brain regions similar to that by the first principal component of $C_{\text{H}}$ (Fig. 7b). These observations suggested that the observed regional effects in subcallosal, entorhinal, parahippocampal and temporal pole regions were the most relevant AD-related contributors to the observed elevated Δ-Age in Fig. 2.

**Figure 10:**
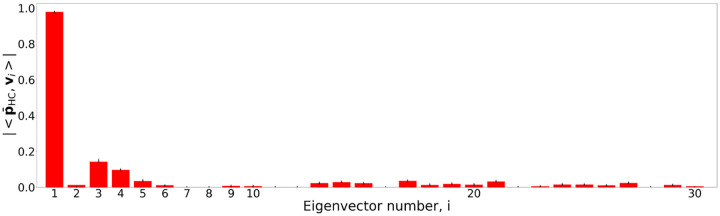
This figure displays a bar plot for $\left|<{\overset{-}{p}}_{\text{HC}},v_{i}\right\rangle |$ for the first 30 eigenvectors of $C_{\text{H}}$ and the vectors of regional contributions, ${\overset{-}{p}}_{\text{HC}}$ in the scenario when VNNs were transferred from XYZ to OASIS-3 dataset.

**Figure 11:**
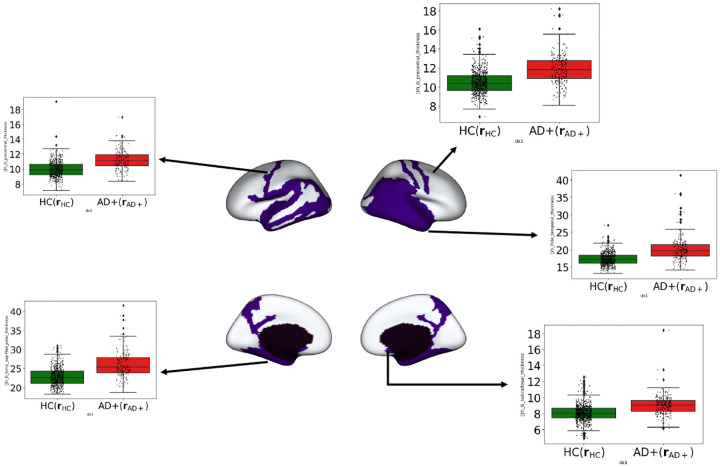
Results depicting the brain regions with significantly elevated regional residuals for AD+ group with respect to HC group in OASIS-3. The results here were derived by a VNN model that was transferred from XYZ to process OASIS-3 and had been trained as a regression model to predict chronological age from cortical thickness data for healthy controls in the XYZ dataset. All shaded regions exhibited significant elevated regional residuals in AD+ with respect to HC (Bonferronicorrected p-value smaller than 0.05).

**Figure 12:**
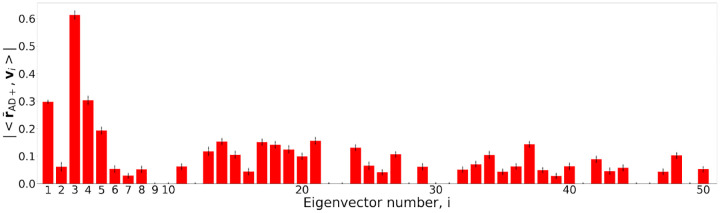
Mean inner product between the normalized vector of regional residuals (norm = 1) of VNN outputs (transferred from XYZ to OASIS-3) obtained from AD+ group and the eigenvectors of $C_{\text{HA}}$ **(covariance matrix of combined HC and AD+ group)**. This figure illustrates a bar plot for $\left|\left\langle {\overset{-}{r}}_{\text{AD}+},v_{i}\right\rangle \right|$ for i∈{1,…,30}, where $v_{i}$ is the i-th eigenvector of covariance matrix $C_{\text{HA}}$ associated with its i-largest eigenvalue. The bars are evaluated from the mean of $\left|\left\langle {\overset{-}{r}}_{\text{AD}+},v_{i}\right\rangle \right|$ obtained for individuals in the AD+ group (results for eigenvectors associated with coefficient of variation of $\left|\left\langle {\overset{-}{r}}_{\text{AD}+},v_{i}\right\rangle \right|$ larger than 30% excluded). For every individual in AD+ group, the association of its regional residuals with eigenvectors of $C_{\text{HA}}$ were evaluated over 100 nominal VNN models (trained on the XYZ dataset).

**Figure 13:**
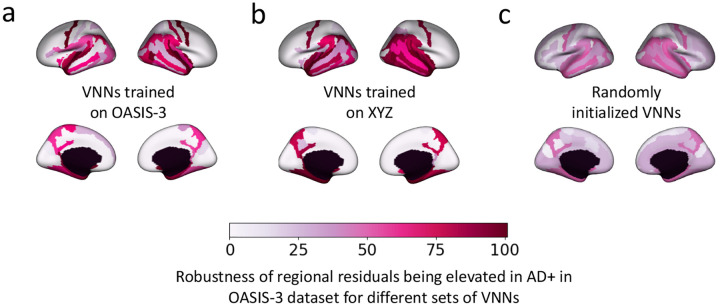
Regional profiles derived from the robustness of elevated regional residuals for AD+ group with respect to HC group using randomly initialized VNNs and VNNs trained to predict chronological age of HC groups in different datasets. Panel a illustrates the robustness of the regional residuals being observed to be elevated for AD+ with respect to HC group across the 100 VNN models that were trained on the OASIS-3 dataset. The number of VNN models for which a brain region was deemed to have a significantly higher regional residual for AD+ group with respect to HC group quantifies the robustness of that brain region as a contributor to the observed elevated Δ-Age in AD+ with respect to HC. Panel **b** displays the regional profiles obtained from the analysis of regional residuals for AD+ and HC groups, where the regional residuals were derived from 100 VNNs trained to predict chronological age for HC group in XYZ. Panel **c** displays the corresponding results derived from 100 VNNs that were randomly initialized.

**Figure 16:**
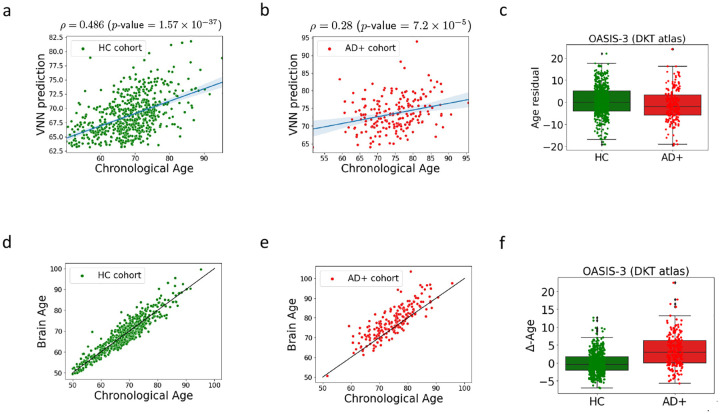
Supplementary figures to Fig. 2. Panel **a** displays the plot of VNN prediction versus chronological age for HC group. VNN predictions were obtained as the average of the outputs of 100 nominal VNNs that were trained on OASIS-3 and operated on the anatomical covariance matrix $C_{\text{HA}}$. Panel **b** displays the results similar to that in panel **a** for the AD+ group. The solid line in panels **a** and **b** is the least squares line. Panel **c** includes the boxplots for residuals derived from the difference between VNN predictions and chronological age for HC and AD+ groups. Panel **d** and **e** display the plots for brain age versus chronological age for HC and AD+ groups, respectively. The solid line in panels **d** and **e** is the identity line. Panel **f** displays the box plots for Δ-Age in HC and AD+ groups.

**Figure 17:**
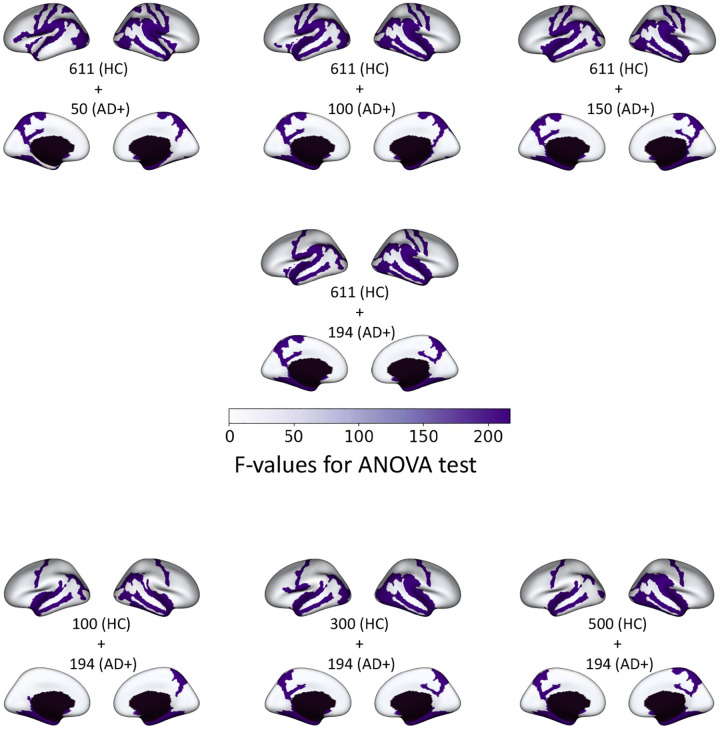
Stability to perturbations in the anatomical covariance matrix for group differences between AD+ and HC groups observed in regional residuals. For a VNN model that was trained to predict chronological age for HC group in OASIS-3 dataset, the regional residuals were first determined using the anatomical covariance matrix $C_{\text{HA}}$ formed by the cortical thickness data of complete OASIS-3 dataset (i.e., 611HC individuals and 194 individuals in the AD+ group. The group differences in regional residuals between AD+ and HC group were investigated according to the procedure in subsection 3.2 In the procedure described therein, we evaluated the F-values for the ANOVA test between regional residuals for AD+ group and HC group. The brain regions associated with the regional residuals that were significantly elevated in AD+ group with respect to HC group are highlighted on the brain template. The stability of the group differences to perturbations in $C_{\text{HA}}$ was further investigated by varying the composition of cortical thickness data from AD+ and HC groups used to estimate $C_{\text{HA}}$. Figures in the top row display the results obtained via analysis of regional residuals by VNNs that processes the cortical thickness data from the complete OASIS-3 dataset over the anatomical covariance matrix $C_{\text{HA}}$ estimated from 611HC individuals and a varying number of individuals from the AD+group. Figures in the bottom row illustrate the results of similar experiments, with the difference that the anatomical covariance matrix $C_{\text{HA}}$ was estimated using all 194 individuals in the AD+ group and varying number of individuals from the HC group. The results corresponding to $C_{\text{HA}}$ that was estimated using 194AD+ individuals and 611HC individuals formed the baseline for comparison for all scenarios.

**Figure 18:**
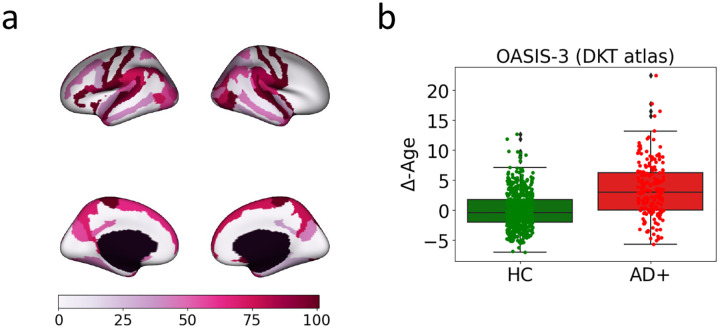
-Age results for anatomical covariance matrix from only HC group $\left({\text{C}}_{\text{H}}\right)$. Δ Panel **a** projects the robustness of observing a significantly higher regional residual for AD+ group with respect to HC group for a brain region on the template. Panel **b** plots the Δ-Age distributions for AD+ and HC groups derived from VNNs with $C_{\text{H}}$ as the covariance matrix.

**Figure 19:**
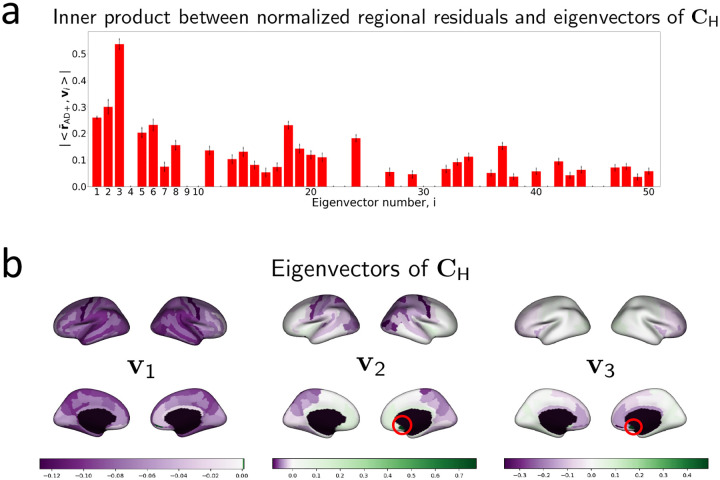
Panel **a** illustrates a bar plot for $\left|\;\left\langle {\overset{-}{r}}_{\text{AD}+},v_{i}\right\rangle \;\right|$ for i∈{1,…,30}, where $v_{i}$ is the i-th eigenvector of covariance matrix $C_{\text{H}}$ associated with its i-largest eigenvalue. The bars are evaluated from the mean of $\left|\;\left\langle {\overset{-}{r}}_{\text{AD}+},v_{i}\right\rangle \right|$ obtained for individuals in the AD+ group (results for eigenvectors associated with coefficient of variation of $\left|\;\left\langle {\overset{-}{r}}_{\text{AD}+},v_{i}\right\rangle \right|$ larger than 30% excluded). For every individual in AD+ group, the association of its regional residuals with eigenvectors of $C_{\text{H}}$ were evaluated over 100 nominal VNN models (trained on the OASIS-3 dataset). The eigenvectors associated with top three largest values for $\left|\;\left\langle {\overset{-}{r}}_{\text{AD}+},v_{i}\right\rangle \;\right|$ are plotted on the brain template in Panel **b**. Subcallosal region in the right hemisphere was associated with the element with the largest magnitude in $v_{2}$ and $v_{3}$ and is highlighted with a red circle in the corresponding plots.

## Data Availability

OASIS-3 dataset is publicly available and hosted on central.xnat.org. Access to OASIS-3 dataset may be requested through https://www.oasis-brains.org/. Code for demonstrating brain age evaluation is available at https://github.com/pennbindlab/VNN_Brain_Age. Requests for details regarding IDs of individuals in OASIS-3 and source data for all figures may be sent to sihags@pennmedicine.upenn.edu

## Appendices

### A Relevant Literature

#### Graph Convolutional Networks.

GCNs typically rely on an information aggregation procedure (referred to as graph convolutions) over a graph structure for data processing. Several implementation strategies for graph convolution operations have been proposed in the literature, including spectral convolutions [46], Chebyshev polynomials [47], ordinary polynomials [29], and diffusion based representations [48]. GCNs admit the properties of stability to topological perturbations and transferability across graphs of different sizes in various settings [30, 49–51], which makes them a well-motivated data analysis tool for graph-structured data.

In [21], coVariance neural networks (VNN) were proposed as GCNs with sample covariance matrices as graph and polynomial graph filters as convolution operation. Covariance matrices and principal component analysis (PCA) form the two cornerstones of non-parametric analyses in real world applications that have spatially distributed, multi-variate data acquisition protocols, including neuroimaging [25], computer vision [52, 53], weather modeling [54], traffic flow analysis [55], and cloud computing [56].

#### Interpretable brain age prediction.

Limited focus has been on comparable studies that associate brain age gaps with regional profiles [16, 57]. The study in [16] adopts a convolutional neural network approach to infer brain age from MRI images directly and assigns importance to brain regions in evaluating the brain age. In principle, the interpretability offered by VNNs in the context of brain age is similar, as we infer a regional profile for Δ-Age by isolating the brain regions that are contributors to the elevated Δ-Age in neurodegeneration. In addition, the regional profile identified by VNNs is correlated with specific eigenvectors or the principal components of the anatomical covariance matrix. Hence, the Δ-Age inferred by our framework is driven by the ability of a VNN to manipulate the input data according to certain principal components of the anatomical covariance matrix. Also, VNNs are significantly less complex deep learning models as compared to those studied in [16]. Our results demonstrate that the VNNs trained with less than 300 learnable parameters exhibit regional interpretability in the context of brain age in AD. In general, the regional expressivity offered by VNNs is in stark contrast to a multitude of existing relevant studies that rely on less transparent statistical approaches and further use post-hoc analyses (such as ablation analysis [58–60] or exploring correlations with region-specific markers [61] and psychiatric symptoms [40, 62]) to assign interpretability to a scalar, elevated Δ-Age effect.

### B An Abstract Overview of VNN-based Brain Age Prediction

Figure 4 provides an abstract overview of the general procedure of evaluating brain age using machine learning (ML) models. From Fig. 4, we note that if the ML model is a black box, it may be infeasible to capture the contributors to elevated age-gap in Step 3. Furthermore, in this context, it is also unclear whether age-bias correction step (Step 2) influences final Δ-Age prediction through some statistical artifact [20]. Hence, it can be desirable to minimize the role of age-bias correction in Δ-Age evaluation by selecting an ML model that achieves a near perfect fit on chronological age of healthy controls in Step 1. However, there is no guarantee that achieving an ‘perfect fit’ on true age of healthy controls will enable the ML model to capture the impact of neurodegeneration in individuals with neurodegeneration.

VNNs allow us to analyze the contribution of each feature (brain region) to the final output. Hence, by analyzing the elevations in contributions of different brain regions via studying group differences in regional residuals, we are able to characterize the brain regions that contribute to accelerated aging (or larger Δ-Age) in individuals with age-related neurodegeneration (Fig. 5). Thus, we can verify that VNNs captured neurodegeneration-driven effects that eventually led to elevated Δ-Age for an individual. Our experiments show that VNNs do not obtain a perfect fit on chronological age of healthy individuals. Hence, age-bias correction is important to appropriately project the VNN model outputs via a linear model into an appropriate space such that a clinician can observe an elevated Δ-Age effect in individuals with neurodegenerative condition (AD in this paper). Based on these observations, we remark that VNNs provide an interpretable framework for brain age prediction.

### D VNN training

We randomly split the HC group into an approximately 90/10 train/test split. Thus, the test set consisted of 61 healthy individuals. The sample covariance matrix was evaluated using all samples in the training set (n=550). Furthermore, this covariance matrix was normalized such that its maximum eigenvalue was 1. Cortical thickness data was z-score normalized across the training set and this normalization was applied to the test set. Next, the training set was randomly split internally, such that, the VNN was trained with respect to the mean squared error loss between the predicted age and the true age in n=489 samples of the HC group. The loss was optimized using batch stochastic gradient descent with Adam optimizer available in PyTorch library [63] for up to 100 epochs. The batch size was 78 (determined via ‘optuna’ package [37]). The VNN model with the best minimum mean squared error performance on the remaining 61 samples in the training set (which acted as a validation set) was included in the set of nominal models for this permutation of the training set. For each dataset, we trained and validated the VNN models over 100 permutations of the complete training set of n=550 samples for the HC group, thus, leading to 100 trained VNN models (also referred to as nominal models) per dataset.

### E VNN regression model outputs for HC group in OASIS-3 are correlated with the first eigenvector of anatomical covariance matrix

The study in [21] suggests that VNN based statistical inference draws conceptual similarities with PCA-driven analysis. Hence, we further investigated whether the regression performance achieved by VNNs in predicting the chronological age of HC group could be characterized by contributions of the eigenvectors of the anatomical covariance matrix. To avoid any selection bias, we report the results on the complete HC group where the cortical thickness features were z-score normalized across the group such that the mean of cortical thickness for a brain region across the HC group was 0. The findings here are also cross-validated using VNNs trained on an independent dataset in Appendix G. Here, the notation $C_{\text{H}}$ denotes the anatomical covariance matrix derived from the complete HC group.

Recall that the final regression output by VNNs is formed by an unweighted average function as a readout function. Thus, we can equivalently represent the functionality of the readout as a simple aggregation of the contributions of different features or brain regions to the final estimate formed by the VNN (see (5)). Hence, for every individual, we evaluated the mean of the inner products (also equivalently referred to as dot product between vectors) between the vectors of contributions of every brain region with the eigenvector of the covariance matrix $C_{\text{H}}$ for all 100VNN models. Note that a vector of regional contributions was of the same length as the number of cortical thickness features (i.e., 148 for OASIS-3) and therefore, each element of this vector was associated with a distinct brain region. We use the notation $p_{\text{HC}}$ to represent the population of vectors obtained from the HC group. To evaluate the inner product, we used ${\overset{-}{p}}_{\text{HC}}$, which was obtained from $p_{\text{HC}}$ after normalization (norm=1). We denote the population of inner products across the HC group in OASIS-3 by $\left|\left\langle {\overset{-}{p}}_{\text{HC}},v_{i}\right\rangle \right|$ for an eigenvector $v_{i}$ of $C_{\text{H}}$. Note that since all vectors in ${\overset{-}{p}}_{\text{HC}}$ were normalized to have norm 1 and the eigenvectors of $C_{\text{H}}$ were of length 1 by default, $\left|\left\langle {\overset{-}{p}}_{\text{HC}},v_{i}\right\rangle \right|$ represented the population of the cosine of the angles between the vectors in $p_{\text{HC}}$ and eigenvectors $v_{i}$ of $C_{\text{H}}$ across the HC group in OASIS-3. Figure 7a plots the mean of the inner products observed across the HC group for the first 30 eigenvectors of $C_{\text{H}}$ for all 100VNNs. Figure 7b illustrates the projection of $v_{1}$ on a brain template.

### F Illustration of regional residual analysis from VNN model outputs

In this section, we demonstrate the regional analysis described in Section 3.2 for a VNN model that was trained to predict chronological age for HC group in OASIS-3 dataset. All mathematical notations referred to in this section are borrowed from Section 3.2. Note that no further training was performed for this VNN model to evaluate brain age or regional residuals.

The covariance matrix in this VNN model was replaced with $C_{\text{HA}}$ derived from the cortical thickness features from both HC and AD+ groups. Further, the cortical thickness features in the HC group were z-score normalized and this normalization was used to transform the cortical thickness features of the AD group.

The age prediction ${\overset{ˆ}{y}}_{i}$ and a vector of residuals $r_{i}$ were obtained for an individual i∈{1,…,805} in the dataset. The size of residual vector $r_{i}$ was 148×1 and hence, each element of $r_{i}$ corresponded to a distinct brain region as defined by the DKT brain atlas with 148 parcellations. By evaluating the vector of residuals $r_{i}$ for every individual in the combined dataset, a population of residual vectors from HC group (referred to as $r_{\text{HC}}$) and AD+ group (referred to as $r_{\text{AD}+}$) was constructed. The elements of these residual vectors are referred to as regional residuals throughout the paper.

Each dimension of these residual vectors was investigated for group differences between HC and AD+ groups via ANOVA as described in Section 3.2. Thus, for every VNN model, we eventually performed m=148 number of ANOVA tests and evaluated the brain regions for significance in group differences in their respective residuals. The significance of group differences between the distributions of regional residuals for HC and AD+ groups corresponding to a brain region was determined after correcting the p-values of ANOVA test for multiple comparisons via Bonferroni correction (Bonferroni corrected p-value < 0.05). The group differences were additionally investigated for significance at an uncorrected level using ANCOVA with age and sex as covariates.

Figure 8 illustrates the results obtained via ANOVA in this context. The brain regions deemed significant according to the criteria provided in Section 3.2 have been shaded. The box plots for various brain regions show that the regional residuals were significantly elevated in AD+ group as compared to HC.

We had 100 trained VNN models for the OASIS-3 dataset and performed similar analyses for each of them. Further, we counted the number of models for which the above described analysis yielded a brain region to be significant. A brain region with robust group difference in its regional residual distribution in HC vs AD+ was expected to be more frequently labeled as significant by the VNN models. The results of this robustness analyses on the OASIS-3 dataset are shown in Fig. 2a.

### G Cross-validation using VNNs trained on an independent dataset

We trained a set of 100VNN models on a healthy population described below.

#### XYZ Dataset.

This dataset consists of the cortical thickness data extracted from healthy individuals (HC; n=105,age=62.6±7.62 years, 57 females). For each individual, the cortical thickness data was curated according to Schaefer atlas [64], at 100 parcel resolution. The ANTs cortical thickness pipeline [65, 66] was used to derive mean cortical thickness within each atlas parcel using 3T T1-weighted MRIs (~1mm isotropic resolution).

The training procedure for the VNNs trained to predict chronological age on the XYZ dataset was similar to that in Section D and is described below.

#### VNN Training.

We randomly split the XYZ dataset into an approximately 90/10 train/test split. Thus, the test set consisted of 10 individuals. The sample covariance matrix was evaluated using all samples in the training set (n=95) and we had the sample covariance matrix $C^{\prime }$ of size m×m(m=100 for XYZ dataset). Furthermore, $C^{\prime }$ was normalized such that its maximum eigenvalue was 1. The cortical thickness features in the training set were z-score normalized and this normalization was applied to the test set. Next, the training set was randomly split internally, such that, the VNN was trained with respect to the mean squared error loss between the predicted age and the true age in n=84 samples. The loss was optimized using batch stochastic gradient descent with Adam optimizer available in PyTorch library [63] for up to 100 epochs. The batch size was 34. The VNN model with the best minimum mean squared error performance on the remaining samples in the training set (which acted as a validation set) was included in the set of nominal models for this permutation of the training set. We trained and validated the VNN models over 100 permutations of the complete training set of n=95 samples, thus, leading to 100 trained VNN models. The hyperparameters for the VNN architecture and learning rate of the optimizer were chosen according to a hyperparameter search procedure using the package Optuna [37]. For this dataset, VNN had a L=2-layer architecture, with a filter bank such that we had F=26 and 2 filter taps in each layer. The learning rate for the optimizer was 0.058. The number of learnable parameters for this VNN was 1456. On the chronological age prediction task, the VNNs achieved the performance of MAE:3.72±0.22 years and Pearson′s correlation: 0.78±0.01 on the test set and MAE:5.39±0.084 years and Pearson′s correlation:0.49±0.017 on the complete XYZ dataset. Thus, the VNNs trained here were not overfit on the training set.

#### Transferring the VNNs from XYZ to OASIS-3.

In general, transferring a VNN model from dataset A to dataset B implies that the VNN was trained for an inference task on dataset A and is being tested for the same inference task on dataset B. The scale-free aspect of VNN architecture allows transferring of VNNs between datasets of different dimensionalities. The transferability aspect of VNNs makes the learned parameters ‘scale-free’ and allowed us to leverage the VNNs trained on XYZ dataset to process OASIS-3. Subsequently, we use the phrase ‘VNNs transferred from XYZ to OASIS-3’ to denote the procedure of using the VNNs trained on XYZ dataset to process OASIS-3. The VNNs transferred from XYZ to OASIS-3 were used to cross-validate the spectral properties of VNNs that were pertinent to generating the regional profile for Δ-Age in Fig. 2a. The observations made in this context enable us to examine whether training the VNNs on a healthy population fine-tuned their parameters in a specific manner that was relevant to inferring Δ-Age. However, note that due to the differences in Schaefer’s atlas and DKT atlas (the former being generated from functional MRI data and the latter being an anatomical atlas), a more nuanced computational approach may be needed to ensure preservation of quantitative performance on the chronological age prediction task when VNNs were transferred between the two datasets. We leave this aspect for future work.

Next, we discuss various aspects of VNN-based brain age prediction in OASIS-3 that were validated by the VNNs that had been trained to predict the chronological age in XYZ dataset.

#### G.1 VNNs trained on XYZ dataset exploited similar eigenvectors of the anatomical covariance matrix as those by VNNs trained on OASIS-3

We first investigated the correlations between the eigenvectors of $C_{\text{H}}$ and the regional contributions derived from VNNs that were transferred from XYZ to HC group in OASIS-3. Similar to Fig. 7a, the first eigenvector of the anatomical covariance matrix $C_{\text{H}}$ had the largest correlation with the regional contributions derived from the VNNs in this setting (0.977±0.0015) and the next two largest associations were observed for the third eigenvector (0.147±0.006) and fourth eigenvector (0.097±0.0027). Figure 10 displays the mean of the inner products observed across the HC group for the first 30 eigenvectors of $C_{\text{H}}$ obtained by the VNNs that were transferred from XYZ to OASIS-3 dataset. Hence, VNNs that were transferred from the XYZ dataset to OASIS-3 dataset leveraged the first eigenvector of $C_{\text{H}}$ to form the chronological age predictions for HC group in OASIS-3 dataset. This observation indicated that the VNNs trained to predict chronological age for the healthy population for one dataset could gain the ability to leverage the relevant eigenvectors of the anatomical covariance matrix when transferred to process another dataset.

Figure 11 illustrates the results obtained by analyzing the regional residuals in the context of brain age, where the regional residuals were derived by transferring a randomly selected VNN model from XYZ dataset to OASIS-3 dataset. Here, we observed that brain regions similar to those in Fig. 8 exhibited significantly elevated regional residuals in the AD+ group. Thus, Fig. 11 provides the evidence that VNN trained on XYZ dataset can transfer the interpretability from XYZ dataset to OASIS-3 dataset.

Given the similarity between Fig. 8 and Fig. 11, it was expected that the regional residuals derived from VNNs that were transferred from XYZ to OASIS-3 exploited the eigenvectors of $C_{\text{HA}}$ in a similar fashion as in Fig. 3. Figure 12 displays the bar plot for the mean of the inner product between the regional residuals for AD+ group and first 50 eigenvectors of $C_{\text{HA}}$.

Similar to Fig. 3, the regional residuals derived from VNNs that were transferred from XYZ to OASIS-3 had the largest correlations with the first, third, and fourth eigenvectors of the anatomical covariance matrix $C_{\text{HA}}\left(\left|<{\overset{-}{r}}_{\text{AD}+},v_{3}>\right|=0.612\pm 0.013,\left|<{\overset{-}{r}}_{\text{AD}+},v_{4}\right\rangle |=0.302\pm 0.014\right.,\left.\left|<{\overset{-}{r}}_{\text{AD}+},v_{1}\right\rangle |=0.296\pm 0.004\right)$. These observations implied that the VNN models trained on XYZ dataset were able to leverage the relevant eigenvectors for brain age prediction in OASIS-3 dataset in the formation of regional residuals. From the findings in Fig. 3 and Fig. 12, we can conclude that the VNN models trained to predict chronological age in OASIS-3 and XYZ datasets were able to leverage the principal components of $C_{\text{HA}}$ that were associated with the regional profile of elevated Δ-Age in Fig. 2.

#### G.2 Regional profiles for brain regions with elevated regional residuals in AD+ group were qualitatively preserved when VNNs are transferred from XYZ to OASIS-3

Finally, we evaluated the regional profile corresponding to the robustness of a brain region exhibiting elevated regional residual in the AD+ group with respect to HC group. This experiment helped determine whether the consistency in correlations with eigenvectors in Fig. 3 and Fig. 12 resulted in qualitative consistency in the regional profiles derived from the VNNs trained on XYZ dataset for regions associated with elevated regional residuals in AD+ group with respect to HC group in OASIS-3. Figure 13b illustrates the projection of the robustness of the observed significantly higher regional residuals for AD+ group with respect to HC group on a brain template, where the robustness was evaluated using 100 nominal VNN models in a similar fashion as for the results in Fig. 2a. For clarity, the regional profile for elevated Δ-Age obtained from the VNNs that were trained on OASIS-3 dataset have been included in Fig. 13a.

The regional profiles obtained using VNNs trained on XYZ dataset in Fig. 13b were largely consistent with that in Fig. 13a This observation implied that the VNNs trained on XYZ dataset possessed the ability to extract regional profiles characteristic of accelerated aging in OASIS-3 dataset. This observation could be attributed to the fact that the VNNs trained on XYZ were able to exploit similar principal components of $C_{\text{HA}}$ as the VNNs that were trained on OASIS-3 dataset.

By training a VNN to predict chronological age in healthy individuals, we aim to gain the information about healthy aging, which is expected to be instrumental in detecting patterns of accelerated aging. Our results thus far have shown that the markers of accelerated aging identified by VNNs trained in this manner can be linked to the principal components of the anatomical covariance matrix irrespective of quantitative transferability. Thus, we hypothesized that training the VNNs to predict chronological age helps fine tune their ability to manipulate the input data using principal components that were relevant to the elevated Δ-Age effect in AD+ group. To further support this hypothesis, we evaluated the regional profiles for brain regions that exhibited elevated regional residuals for AD+ group with respect to HC group in OASIS-3 using 100 VNNs that were randomly initialized (i.e., not trained whatsoever) and had the same architecture as the VNNs that were trained on OASIS-3. Figure 13c demonstrates the regional profiles derived from randomly initialized VNNs on OASIS-3 dataset. Figure 13c shows the robustness of the elevated regional residuals for AD+ group with respect to HC group was severely diminished with respect to the parallel results in Fig. 13a and Fig. 13b.

Our experiments here have demonstrated that the ability of VNNs to extract information about accelerated aging from cortical thickness data relies on their ability to exploit the eigenvectors or principal components of $C_{\text{HA}}$ and not necessarily the performance on the task of predicting chronological age of healthy individuals. Thus, training the VNNs to predict chronological age in HC group helped fine tune their parameters to exploit the eigenvectors or principal components of $C_{\text{HA}}$ relevant to Δ-Age in AD+ group. The observations above facilitate the decoupling of the task of brain age prediction from the objective of achieving near perfect performance on the task of chronological age prediction in the HC group.

### H Additional details on brain age prediction in OASIS-3

In this section, we provide additional figures and discussions pertaining to the results for interpretable brain age prediction in Fig. 2. Figure 14 displays the distributions of chronological age for AD+ and HC groups in OASIS-3 dataset.

Since VNNs were trained for the regression task, a VNN processed cortical thickness data and provided an estimate for the chronological age for each individual. Since we trained 100VNN models on different permutations of the training set in OASIS-3, we use the mean of all VNN estimates as the VNN prediction for an individual. This VNN prediction is further leveraged to form brain age estimates and Δ-Age metrics. Figure 16a displays the plot for VNN predictions versus chronological age (ground truth) for the complete HC group. The Pearson’s correlation between VNN prediction and chronological age (ground truth) for HC group was 0.486, which was similar to that reported in Section 4.1 VNN outputs clearly under-estimated the chronological age for older individuals and over-estimated the chronological age for individuals on the younger end of the age distribution for HC group.

Figure 16b displays the plot for VNN predictions versus chronological age (ground truth) for the complete AD+ group. The Pearson’s correlation between VNN prediction and chronological age (ground truth) for AD+ group was 0.28. We further note that the VNN architecture and our analysis of regional residuals helped quantify the contribution of each brain region to a data point in Fig. 16a and Fig. 16b. Hence, the scatter plot in Fig. 16b could be affected by larger contributions of certain brain regions for AD+ group relative to the HC group.

Figure 16c illustrates the box plots of residuals evaluated by the difference between VNN predictions and chronological age for HC and AD+ groups. Figure 16c suggests that the chronological age for AD+ group was underestimated as compared to that for HC group. This observation was also expected since AD+ group is significantly older than the HC group. However, we expect that the robust elevated regional residuals from brain regions in Fig. 2b mitigated the under-estimation effect due to higher age of AD+ group to some extent.

Figures 16d–f display the results after age-bias correction is applied to the VNN outputs. As expected, the brain age for HC group in Fig. 16d is largely concentrated around the line of equality (x=y line). In contrast, the brain age for AD+ group in Fig. 16e is concentrated above the line of equality. These effects manifest into the box plots for Δ-Age in Fig. 16f where we observe the AD+ group to have elevated Δ-Age as compared to HC group.

VNN architecture facilitated isolation of the effects of accelerated aging before age-bias correction was applied. Hence, the transformation of VNN outputs to brain age from Fig. 16a–c to Fig. 16d–f was not surprising. However, such insights may be infeasible for machine learning approaches that lack transparency and hence, the impact of deviations due to neurodegeneration from the healthy control population cannot be interpreted or isolated. In this context, if the learning model was a black box, Fig. 16a–c may appear to be counter-intuitive to the goal of detecting accelerated aging in the AD+ group and the effect of age-bias correction can be unclear, thus, leading to several criticisms [20].

### I Adaptive readouts may penalize the interpretability of regional residuals and Δ-Age

Thus far, we have focused on VNNs that operate with a non-adaptive readout (unweighted average) function. However, it is expected that the performance of the VNNs on their original task of chronological age prediction could be improved significantly with the help of an adaptive readout function. Our experiments showed that this was indeed the case. If a single-layer fully connected perceptron consisting of 10 neurons was added to the VNNs with the same architecture as the ones that were trained on OASIS-3 dataset, we could improve the performance on the chronological prediction task. For 100VNNs with adaptive readout that were trained on random permutations of the training data, the median MAE for the HC group was 4.64 years, which was significantly smaller than the MAE achieved by VNNs with non-adaptive readouts (Section 4.1). Among the 100 VNN models with adaptive readouts, we analyzed the regional residuals for one VNN model with adaptive readout that had the best performance on chronological age prediction in HC group (test set: MAE = 4.17 years, Pearson’s correlation between prediction and ground truth = 0.73; complete HC group: MAE=4.26 years, Pearson’s correlation between prediction and ground truth = 0.725). Our regional residuals revealed no significant difference between the regional residuals for AD+ group and HC group. This observation suggested that VNN lost its interpretability due to the addition of adaptive readout function. Moreover, we also observed a diminished gap between Δ-Age for AD+ and HC groups determined using this VNN model (Δ-Age for AD group: 1.58±4.67 years, Δ-Age for HC group: 0±3.45 years, Cohen′s d=0.384). The findings discussed here suggest that boosting the performance on chronological age prediction task by using an adaptive readout function may penalize the interpretability offered by VNNs with non-adaptive readouts and also diminish the Δ-Age gap between AD+ and HC groups.

### J Regional profiles corresponding to elevated regional residuals in AD+ group are stable to the composition of data used to estimate anatomical covariance matrix $C_{\text{HA}}$

Recall that Δ-Age and associated regional profiles were evaluated using VNNs that operated upon a composite anatomical covariance matrix $C_{\text{HA}}$. We next checked whether the results derived from VNNs relevant to Δ-Age were stable to the changes in composition of the combined HC and AD+ groups used to estimate the anatomical covariance matrix. Note that the bilateral parahippocampal, entorhinal, subcallosal, and temporal pole regions are expected to be among the most relevant to Δ-Age in AD based on the results in Fig. 9.

We performed two sets of experiments. In the first set, we included the whole HC group and gradually varied the number of individuals from the AD+ group to be included to form the estimate ${\text{C}}_{\text{HA}}$. Figure 17a includes the results obtained from a randomly selected VNN model corresponding to the anatomical covariance matrix formed by different combinations of the individuals from HC and AD+ groups. The results in Fig. 17a display the brain regions whose regional residuals from AD+ group were higher than that in the HC group (Bonferroni corrected p-value < 0.05). The result obtained by the VNN when it used $C_{\text{HA}}$ estimated from all 611HC individuals and 194 AD+ individuals forms the baseline to evaluate stability in this context. When the covariance matrix $C_{\text{HA}}$ was perturbed by using a smaller number of individuals from the AD+ group to estimate it, we observed that the significance of the relevant brain regions (parahippocampal, subcallosal and temporal pole) were preserved till exclusion of about 144AD+ individuals from the estimate $C_{\text{HA}}$. Hence, the significant differences observed in the regional residuals for AD+ and HC groups in the aforementioned regions were robust to perturbations in $C_{\text{HA}}$ due to variability in the number of individuals from the AD+ group.

Figure 17b illustrates the results obtained for a similar experiment as above, with the difference that the regional residuals were evaluated for the VNN when the anatomical covariance matrix $C_{\text{HA}}$ was perturbed by reducing the number of individuals from the HC group used to estimate it. Using the result obtained for $C_{\text{HA}}$ estimated from 611HC individuals and 194AD+ individuals in Fig. 17a as the baseline, the results pertaining to ANOVA between regional residuals for AD+ and HC groups (with AD+ elevated as compared to HC) remained consistent as long as 100 or more individuals from HC group were included in forming $C_{\text{HA}}$. With less than 100 number of HC individuals included in ${\text{C}}_{\text{HA}}$, the results became noticeably less significant in precuneus and supramarginal regions in the left hemisphere.

In summary, the group differences observed between the regional residuals for AD+ and HC groups in OASIS-3 dataset were robust to perturbations in the covariance matrix $C_{\text{HA}}$ when it was perturbed from the baseline by using a different combination of HC and AD+ individuals to estimate it. However, we also remark that (nearly) complete exclusion of HC or AD+ groups from $C_{\text{HA}}$ resulted in loss of significance of the elevation in regional residuals in AD+ for various regions, including bilateral parahippocampal and temporal pole regions, and precuneus and supramarginal regions in the left hemisphere. Thus, both HC and AD+ groups were relevant to the anatomical covariance matrix $C_{\text{HA}}$ that resulted in regional profiles in Fig. 2a, and they were robust to the combination of individuals from HC and AD+ used to estimate $C_{\text{HA}}$.

### K Δ-Age evaluation with anatomical covariance matrix from HC group

Using the anatomical covariance matrix derived only from the HC group (denoted by $C_{\text{H}}$) resulted in observations that were consistent with Fig. 2 with a slightly diminished difference in Δ-Age between HC and AD+ groups. Specifically, Δ-Age for AD+ group in this setting was 3.41±4.57 years, which was significantly larger than that for the HC group (ANOVA: partial $\eta^{2}=0.141,\text{Cohen}\prime \text{s }d=0.88$). The correlation between Δ-Age and CDR sum of boxes scores in the AD+ group was 0.464. Thus, when the anatomical covariance matrix derived solely from the HC group was utilized in our brain age prediction framework, the magnitude of Δ-Age and its utility as a marker of dementia severity was slightly diminished as compared to the results in Fig. 2.

Figure 18a displays the regional profile associated with Δ-Age derived from VNNs with $C_{\text{H}}$ as the anatomical covariance matrix. Comparison with Fig. 2a reveals that the robustness of regional residuals being elevated in the AD+ group in subcallosal and temporal regions was preserved, but that in bilateral entorhinal and parahippocampal regions was diminished in this scenario. Figure 18b displays the distributions for Δ-Age in AD+ and HC groups.

The variation in the regional residuals associated with entorhinal and parahippocampal regions in Fig. 18a and Fig. 2a could be explained by investigating the eigenvectors of $C_{\text{H}}$. Specifically, Fig. 19a displays the associations between regional residuals for the AD+ group and the first 50 eigenvectors of $C_{\text{H}}$, where we observed that the third, second, and first eigenvectors of $C_{\text{H}}$ had the top three largest associations. Figure 19b plots the projections of the first three eigenvectors of $C_{\text{H}}$ on a brain template. Note that the eigenvectors $v_{3}$ and $v_{2}$ have the largest weights associated with the subcallosal region in the right hemisphere, which is consistent with the relevant eigenvectors of $C_{\text{HA}}$ in Fig. 9. However, unlike the eigenvectors of $C_{\text{H}}$ in Fig. 19b, the eigenvectors of $C_{\text{HA}}$ in Fig. 9b are also characterized by comparatively larger weights in the entorhinal and parahippocampal regions. Since parapippocampal and entorhinal regions are well known to be associated with disease onset and cortical atrophy [67], it can be concluded that the anatomical covariance matrix $C_{\text{HA}}$ provides a more holistic perspective to Δ-Age in AD.
