# Supplementary material for: Explainable Brain Age Prediction using coVariance Neural Networks
Source: ArXiv. 2023 Oct 27:arXiv:2305.18370v3. Preprint. [Version 3] (PMC10557794)
Supplement: 1 [file NIHPP2305.18370V3-supplement-1.pdf]

A Data and Code Availability

OASIS-3 dataset is publicly available and hosted on `central.xnat.org`. Access to OASIS-3 dataset may be requested through `https://www.oasis-brains.org/`. Supplementary files include (i) the VNN models trained to predict chronological age for HC group, (ii) code for demonstrations of evaluating regional profiles corresponding to elevated regional residuals in AD+ group, and correlations between the eigenvectors of the anatomical covariance and VNN outputs using a small subset of z-score normalized cortical thickness data, (iii) the regional residuals derived from different VNN models for the OASIS-3 dataset that lead to results in Fig. 2, and (iv) the code for brain age evaluation on OASIS-3 dataset. All material is also available online athttps://github.com/sihags/VNN_Brain_Age.

B Relevant Literature

Graph convolutional networks. GCNs typically rely on an information aggregation procedure (referred to as graph convolutions) over a graph structure for data processing. Several implementation strategies for graph convolution operations have been proposed in the literature, including spectral convolutions [53], Chebyshev polynomials [54], ordinary polynomials [36], and diffusion based representations [55]. GCNs admit the properties of stability to topological perturbations and transferability across graphs of different sizes in various settings [38, 56–58], which makes them a well-motivated data analysis tool for graph-structured data.

In [31], coVariance neural networks (VNN) were proposed as GCNs with sample covariance matrices as graph and polynomial graph filters as convolution operation. Covariance matrices and principal component analysis (PCA) form the two cornerstones of non-parametric analyses in real world applications that have spatially distributed, multi-variate data acquisition protocols, including neuroimaging [19], computer vision [59, 60], weather modeling [61], traffic flow analysis [62], and cloud computing [63].

Explainability in GNNs. We refer the reader to [32, 64] for a detailed review on explainability in GNNs. Here, we adopt the taxonomy from [32], which categorizes the recent efforts to add explainability to GNN models into two categories: instance-level methods and model-level methods for explainability. Instance-level methods for explainability are extensions of the standard model-agnostic methods for wider categories of deep learning models to GNNs, and aim to identify features most important to the inference outcome. Examples of techniques used to determine the importance of features include gradient-based methods [65], perturbation-based methods [66], and surrogate methods [67]. Such methods are, in principle, input-dependent as they provide explanations based on the given instance of the dataset. The robustness of the conclusions drawn from such methods to perturbations in the input and variations in the model training algorithms is an active area of research.

Model-level explainability has previously been studied in terms of graph topology in [68]. Further, importance of subgraphs to inference outcomes using Shapley values was studied in [69]. Since graph topology informs convolution operations in GNNs, it can provide a more generic explanation to GNNs than those that focus on individual features (such as nodes or edges of the graph). Since the convolution operation in VNNs is equivalent to manipulating the input data according to the eigenvectors of the covariance matrix [31], the eigenvectors of the covariance matrix are instrumental to explaining the inference by VNNs. Hence, we argue that VNNs can offer model-level explainability, similar in spirit as discussed in [32], where the explainability hinges on the eigenvectors of the covariance matrix.

Interpretable brain age prediction. Limited focus has been on comparable studies that associate brain age gaps with regional profiles [20, 70]. The study in [20] adopts a convolutional neural network approach to infer brain age from MRI images directly and assigns importance to brain regions in evaluating the brain age. In principle, the interpretability offered by VNNs in the context of brain age is similar, as we infer a regional profile for Δ-Age by isolating the brain regions that are contributors to the elevated Δ-Age in neurodegeneration. In addition, the regional profile identified by VNNs is correlated with specific eigenvectors or the principal components of the anatomical covariance matrix. Hence, the Δ-Age inferred by our framework is driven by the ability of a VNN to manipulate the input data according to certain principal components of the anatomical covariance matrix. Also, VNNs are significantly less complex deep learning models as compared to those studied in [20]. Our results demonstrate that the VNNs trained with less than 300 learnable parameters

exhibit regional interpretability in the context of brain age in AD. In general, the regional expressivity offered by VNNs is in stark contrast to a multitude of existing relevant studies that rely on less transparent statistical approaches and further use post-hoc analyses (such as ablation analysis [71–73] or exploring correlations with region-specific markers [25] and psychiatric symptoms [47, 74]) to assign interpretability to a scalar, elevated Δ-Age effect.

C An Abstract Overview of VNN-based Brain Age Prediction

Figure 4 provides an abstract overview of the general procedure of evaluating brain age using machine learning (ML) models. From Fig. 4, we note that if the ML model is a black box, it may be infeasible to capture the contributors to elevated age-gap in Step 3. Furthermore, in this context, it is also unclear whether age-bias correction step (Step 2) influences final Δ-Age prediction through some statistical artifact [23]. Hence, it can be desirable to minimize the role of age-bias correction in Δ-Age evaluation by selecting an ML model that achieves a near perfect fit on chronological age of healthy controls in Step 1. However, there is no guarantee that achieving an 'perfect fit' on true age of healthy controls will enable the ML model to capture the impact of neurodegeneration in individuals with neurodegeneration.

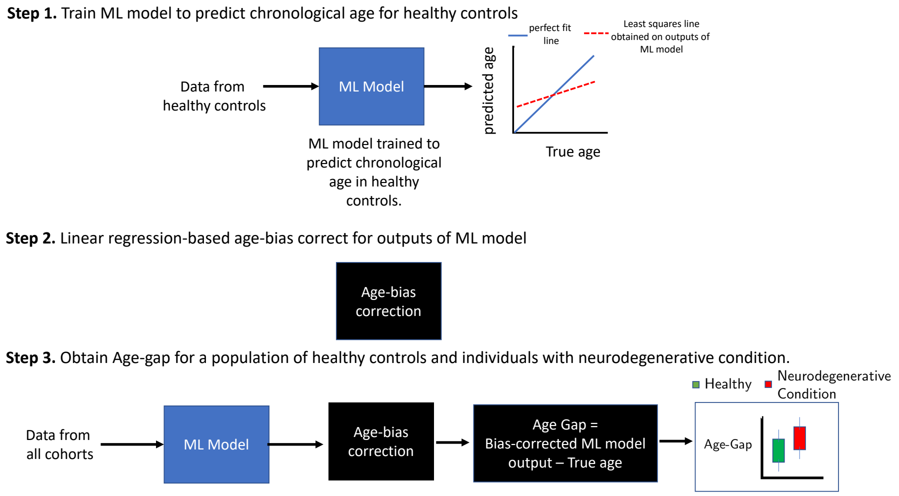

Figure 4: **A general overview of brain age evaluation using machine learning algorithms in the existing literature. Step 1** consists of training a machine learning (ML) model to predict chronological age (true age) for healthy controls. If the correlation between predicted age and true age is smaller than 1, an age-bias exists in ML model outputs as the age for older individuals tends to be under-estimated and that for younger individuals tends to be over-estimated. To correct for this bias, a linear regression based model is applied on the ML model outputs in **Step 2**. Under the hypothesis that ML model can capture accelerated aging in neurodegeneration, it is expected that Δ-Age for individuals with neurodegeneration will be significantly higher than those of healthy controls (**Step 3**).

VNNs allow us to analyze the contribution of each feature (brain region) to the final output. Hence, by analyzing the elevations in contributions of different brain regions via studying group differences in regional residuals, we are able to characterize the brain regions that contribute to accelerated aging (or larger Δ-Age) in individuals with neurodegeneration (Fig. 5). Thus, we can verify that VNNs captured neurodegeneration-driven effects that eventually led to elevated Δ-Age for an individual. Our experiments show that VNNs do not obtain a perfect fit on chronological age of healthy individuals. Hence, age-bias correction is important to appropriately project the VNN model outputs via a linear model into an appropriate space such that a clinician can observe an elevated Δ-Age effect in individuals with neurodegenerative condition (AD in this paper). Based on these observations, we remark that VNNs provide an interpretable framework for brain age prediction.

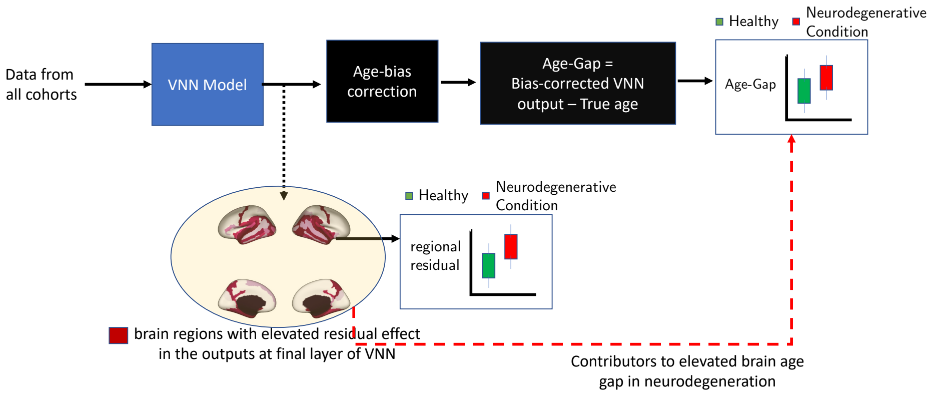

Figure 5: **Interpretability offered by VNNs in brain age prediction.** By analyzing the final layer outputs of VNNs, we can isolate brain regions that have larger regional residuals for individuals with AD with respect to healthy controls. Furthermore, the elevated regional residuals in these brain regions eventually contribute to elevated Δ-Age after age-bias correction.

D VNN Architecture

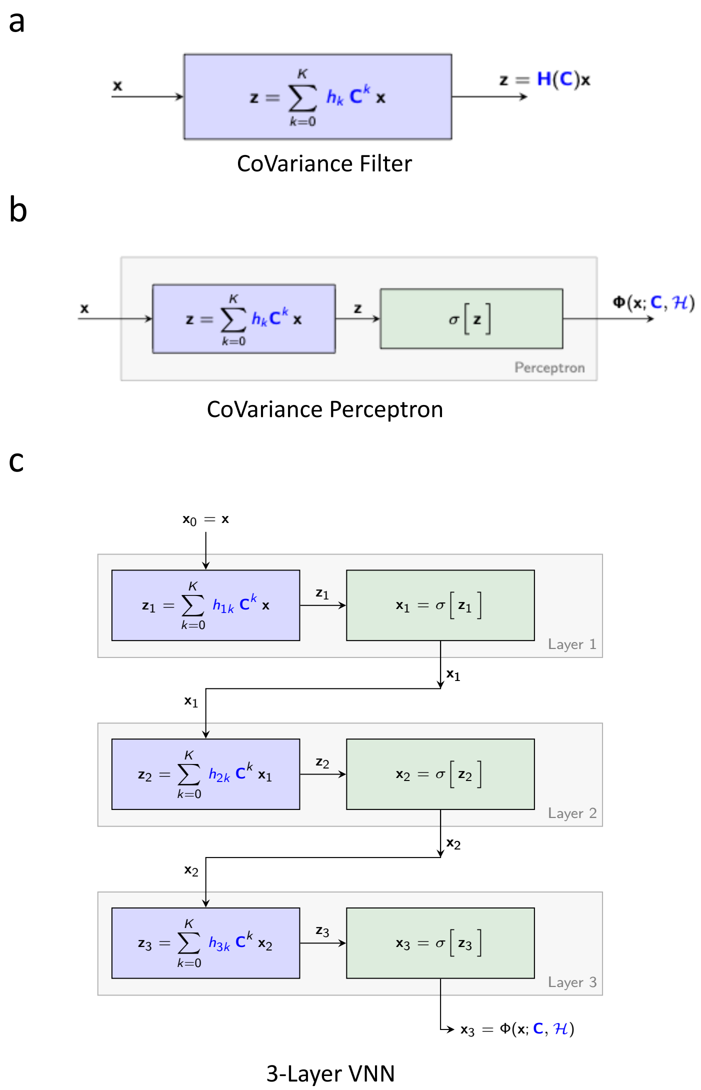

Figure 6: **Basics of VNN architecture.** Panel **a** illustrates that the coVariance filter $\mathbf{H}(\mathbf{C})$ is a polynomial in $\mathbf{C}$ and its application on input $\mathbf{x}$. Panel **b** shows the construction of a coVariance perceptron based on coVariance filter $\mathbf{H}(\mathbf{C})$ and pointwise nonlinearity σ. coVariance perceptron specifies one layer of VNN. Panel **c** shows a basic multi-layer VNN architecture formed by stacking three coVariance perceptrons.

E VNN training

We randomly split the HC group into an approximately $90/10$ train/test split. Thus, the test set consisted of 61 healthy individuals. The sample covariance matrix was evaluated using all samples in the training set ($n = 550$). Furthermore, this covariance matrix was normalized such that its maximum eigenvalue was 1. Cortical thickness data was z-score normalized across the training set and this normalization was applied to the test set. Next, the training set was randomly split internally, such that, the VNN was trained with respect to the mean squared error loss between the predicted age and the true age in $n = 489$ samples of the HC group. The loss was optimized using batch stochastic gradient descent with Adam optimizer available in PyTorch library [75] for up to 100 epochs. The batch size was 78 (determined via 'optuna' package [44]). The VNN model with the best minimum mean squared error performance on the remaining 61 samples in the training set (which acted as a validation set) was included in the set of nominal models for this permutation of the training set. For each dataset, we trained and validated the VNN models over 100 permutations of the complete training set of $n = 550$ samples for the HC group, thus, leading to 100 trained VNN models (also referred to as nominal models) per dataset.

F VNN regression model outputs for HC group in OASIS-3 are correlated with the first eigenvector of anatomical covariance matrix

The study in [31] suggests that VNN based statistical inference draws conceptual similarities with PCA-driven analysis. Hence, we further investigated whether the regression performance achieved by VNNs in predicting the chronological age of HC group could be characterized by contributions of the eigenvectors of the anatomical covariance matrix. To avoid any selection bias, we report the results on the complete HC group, where the cortical thickness features were z-score normalized across the group such that the mean of cortical thickness for a brain region across the HC group was 0. Here, the notation $\mathbf{C}_{\mathsf{H}}$ denotes the anatomical covariance matrix derived from the complete HC group.

Recall that the final regression output by VNNs is formed by an unweighted average function as a readout function. Thus, we can equivalently represent the functionality of the readout as a simple aggregation of the contributions of different features or brain regions to the final estimate formed by the VNN (see (5)). Hence, for every individual, we evaluated the mean of the inner products (also equivalently referred to as dot product between vectors) between the vectors of contributions of every brain region with the eigenvector of the covariance matrix $\mathbf{C}_{\mathsf{H}}$ for all 100 VNN models. Note that a vector of regional contributions was of the same length as the number of cortical thickness features (i.e., 148 for OASIS-3) and therefore, each element of this vector was associated with a distinct brain region. We use the notation $\mathbf{p}_{\mathsf{HC}}$ to represent the population of vectors obtained from the HC group. To evaluate the inner product, we used $\bar{\mathbf{p}}_{\mathsf{HC}}$, which was obtained from $\mathbf{p}_{\mathsf{HC}}$ after normalization (norm = 1). We denote the population of inner products across the HC group in OASIS-3 by $|<\bar{\mathbf{p}}_{\mathsf{HC}}, \mathbf{v}_i>|$ for an eigenvector $\mathbf{v}_i$ of $\mathbf{C}_{\mathsf{H}}$. Note that since all vectors in $\bar{\mathbf{p}}_{\mathsf{HC}}$ were normalized to have norm 1 and the eigenvectors of $\mathbf{C}_{\mathsf{H}}$ were of length 1 by default, $| < \bar{\mathbf{p}}_{\mathsf{HC}}, \mathbf{v}_i > |$ represented the population of the cosine of the angles between the vectors in $\mathbf{p}_{\mathsf{HC}}$ and eigenvectors $\mathbf{v}_i$ of $\mathbf{C}_{\mathsf{H}}$ across the HC group in OASIS-3. Figure 7a plots the mean of the inner products observed across the HC group for the first 30 eigenvectors of $\mathbf{C}_{\mathsf{H}}$ for all 100 VNNs. Figure 7b illustrates the projection of $\mathbf{v}_1$ on a brain template.

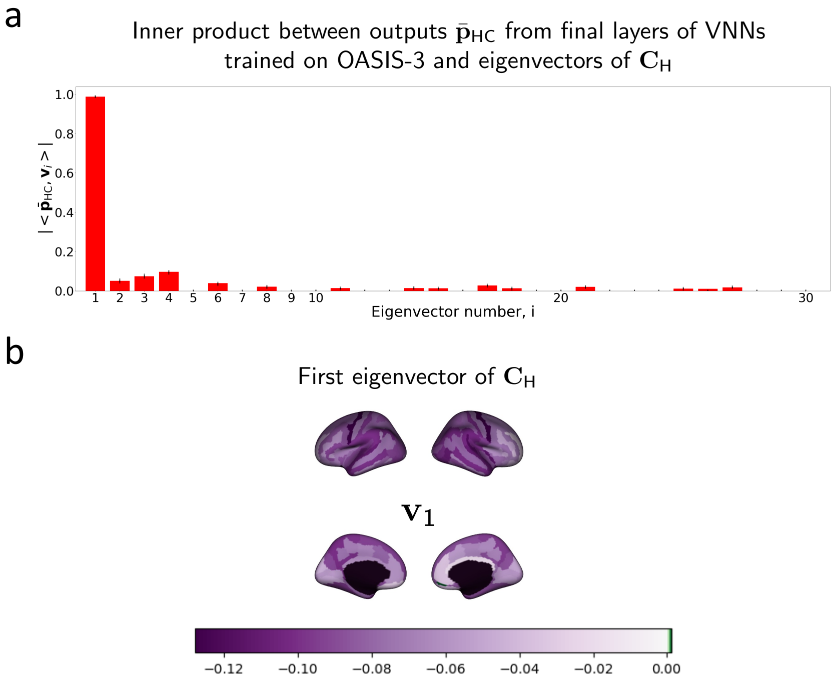

Figure 7: **Inner product between the normalized vector of regional contributions to the VNN outputs ($\bar{\mathbf{p}}_{\mathsf{HC}}$) and eigenvectors of C_{H} (anatomical covariance matrix for HC group in OASIS-3).** Panel **a** illustrates a bar plot for $|<\bar{\mathbf{p}}_{\mathsf{HC}}, \mathbf{v}_i>|$ for $i \in \{1, \ldots, 30\}$, where $\mathbf{v}_i$ is the i-th eigenvector (principal component) of covariance matrix $\mathbf{C}_{\mathsf{H}}$ and associated with i-largest eigenvalue in terms of magnitude and the vectors of regional contributions, $\bar{\mathbf{p}}_{\mathsf{HC}}$ were obtained by VNNs that were trained on OASIS-3 dataset. The inner product results for eigenvectors with coefficient of variation greater than 30% across the HC group of OASIS-3 were excluded (and hence, their respective entries set as 0). For every individual in HC group, the associations between their corresponding vector of regional contributions, $\bar{\mathbf{p}}_{\mathsf{HC}}$ and eigenvectors of $\mathbf{C}_{\mathsf{H}}$ were evaluated over 100 nominal VNN models. The first eigenvector ($\mathbf{v}_1$) had the largest association. The eigenvector $\mathbf{v}_1$ is plotted on a brain template in Panel **b**.

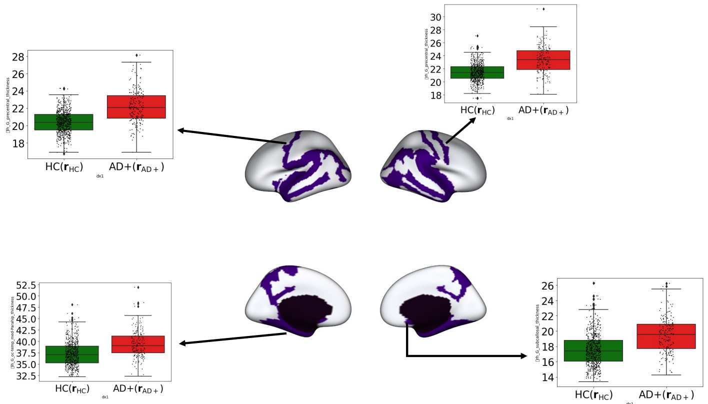

Figure 8: Results depicting the brain regions with significantly elevated regional residuals for AD+ group with respect to HC group in OASIS-3. The results here were derived by a VNN model that was trained as a regression model to predict chronological age from cortical thickness data for HC group in OASIS-3. Box plots depicting the distributions of regional residuals in the HC and AD+ groups are shown for a few representative regions.

G Illustration of regional residual analysis from VNN model outputs

In this section, we demonstrate the regional analysis described in Section 3.2 for a VNN model that was trained to predict chronological age for HC group in OASIS-3 dataset. All mathematical notations referred to in this section are borrowed from Section 3.2. Note that no further training was performed for this VNN model to evaluate brain age or regional residuals.

The covariance matrix in this VNN model was replaced with $\mathbf{C}_{\mathsf{HA}}$ derived from the cortical thickness features from both HC and AD+ groups. Further, the cortical thickness features in the HC group were z-score normalized and this normalization was used to transform the cortical thickness features of the AD group.

The age prediction $\hat{y}_i$ and a vector of residuals $\mathbf{r}_i$ were obtained for an individual $i \in \{1, \ldots, 805\}$ in the dataset. The size of residual vector $\mathbf{r}_i$ was 148×1 and hence, each element of $\mathbf{r}_i$ corresponded to a distinct brain region as defined by the DKT brain atlas with 148 parcellations. By evaluating the vector of residuals $\mathbf{r}_i$ for every individual in the combined dataset, a population of residual vectors from HC group (referred to as $\mathbf{r}_{\mathsf{HC}}$) and AD+ group (referred to as $\mathbf{r}_{\mathsf{AD+}}$) was constructed. The elements of these residual vectors are referred to as regional residuals throughout the paper.

Each dimension of these residual vectors was investigated for group differences between HC and AD+ groups via ANOVA as described in Section 3.2. Thus, for every VNN model, we eventually performed $m = 148$ number of ANOVA tests and evaluated the brain regions for significance in group differences in their respective residuals. The significance of group differences between the distributions of regional residuals for HC and AD+ groups corresponding to a brain region was determined after correcting the p-values of ANOVA test for multiple comparisons via Bonferroni correction (Bonferroni corrected p-value < 0.05). The group differences were additionally investigated for significance at an uncorrected level using ANCOVA with age and sex as covariates.

Figure 8 illustrates the results obtained via ANOVA in this context. The brain regions deemed significant according to the criteria provided in Section 3.2 have been shaded. The box plots for various brain regions show that the regional residuals were significantly elevated in AD+ group as compared to HC. The regional residuals that lead to the results in Fig. 8 are provided in the supplementary material (model ID 50) as part of the regional residuals extracted from all 100 VNN models that were trained on HC group in OASIS-3.

We had 100 trained VNN models for the OASIS-3 dataset and performed similar analyses for each of them. Further, we counted the number of models for which the above described analysis yielded a brain region to be significant. A brain region with robust group difference in its regional residual distribution in HC vs AD+ was expected to be more frequently labeled as significant by the VNN models. The results of this robustness analyses on the OASIS-3 dataset are shown in Fig. 2a.

H Cross-validation on ADNI-1 dataset

In this section, we provide results on the standardized 3.0 T ADNI1 dataset (see [34] for details), consisting of 47 controls (age = 75.06 ± 3.93 years, 29 females), 71 individuals with mild cognitive impairment (age = 74.03 ± 8.12 years, 26 females), and 33 individuals with dementia (age = 75.08 ± 8.07 years, 22 females) from the from the wider ADNI database. We chose this standardized dataset in the spirit of reproducibility and to avoid selection bias. The individuals with mild cognitive impairment and dementia diagnosis were combined to form the AD+ cohort, equivalent to that of the AD+ cohort for OASIS-3.

Data used from ADNI database were obtained from the Alzheimer's Disease Neuroimaging Initiative (ADNI) database. The ADNI was launched in 2003 as a public-private partnership, led by Principal Investigator Michael W. Weiner, MD. The primary goal of ADNI has been to test whether serial magnetic resonance imaging (MRI), positron emission tomography (PET), other biological markers, and clinical and neuropsychological assessment can be combined to measure the progression of mild cognitive impairment (MCI) and early Alzheimer's disease (AD). For up-to-date information, see www.adni-info.org.

Data processing. The MRI images for the 3.0T standardized ADNI-1 dataset at the baseline visit were downloaded from https://adni.loni.usc.edu/. Cortical thickness features (curated according to DKT atlas) were derived using the open-access CAT12 pipeline [76] using their default options. We refer the reader to https://neuro-jena.github.io/cat12-help/ for detailed processing steps. All outputs were quality checked visually for errors in grey matter segmentation.

Cross-validation. The results in Fig. 9 were obtained from the models that were trained on OASIS-3 dataset. Here, the anatomical covariance matrix was estimated using cortical thickness measures from all individuals in ADNI1 dataset. The observations on ADNI1 were highly consistent with those made in the paper. Specifically, in ADNI1 dataset, brain age gap was significantly higher for individuals with dementia as compared to healthy controls, with MCI in between them. Also, the subcallosal, entorhinal, temporal pole, and superior temporal regions were cross-validated on this dataset as being contributors to elevated Δ-Age in the combined cohort of dementia and MCI (equivalent to AD+ group from OASIS-3) with respect to the HC group.

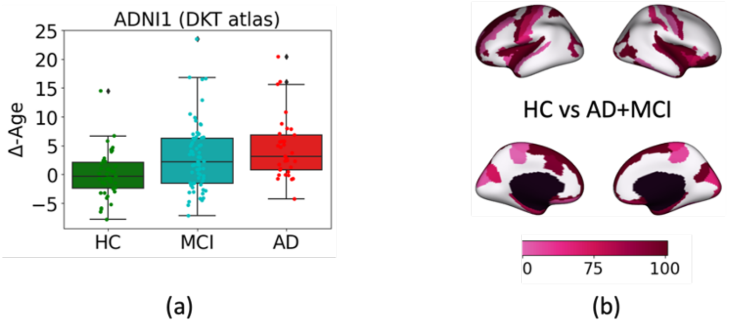

Figure 9: (a) Distribution of Δ-Age across HC, MCI, Dementia cohorts derived from VNN models trained on OASIS-3. Anatomical covariance matrix from ADNI-1 dataset was used in the VNNs. Δ-Age for controls: 0 ± 3.92 years, Δ-Age for MCI 3 ± 5.74 years, Δ-Age for AD: 4.49 ± 5.34 years. (b) Across 100 VNNs that had been trained on OASIS-3 dataset, we evaluated the number of times the regional residual mean was smaller for HC group than the AD+MCI group in ADNI-1 dataset.

I Additional details on brain age prediction in OASIS-3

In this section, we provide additional figures and discussions pertaining to the results for interpretable brain age prediction in Fig. 2. Figure 10 displays the distributions of chronological age for AD+ and HC groups in OASIS-3 dataset.

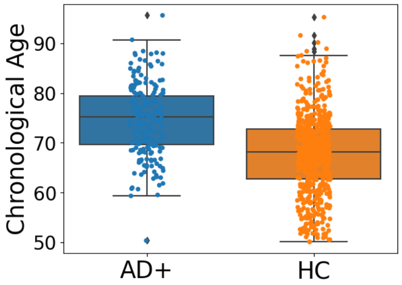

Figure 10: Distribution of chronological age in AD+ and HC groups.

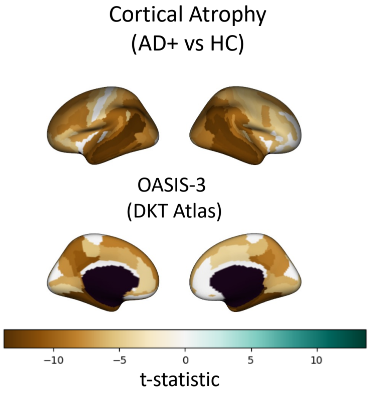

Figure 11: Results of group differences in cortical thickness between AD+ and HC groups. Regions with significant differences (two-sided t-test, Bonferroni corrected p-value < 0.05) are identified and the corresponding t-statistics are projected on a brain template. Negative t-statistic for a brain region suggests that the AD+ group had significant cortical atrophy in that region as compared to HC group.

Since VNNs were trained for the regression task, a VNN processed cortical thickness data and provided an estimate for the chronological age for each individual. Since we trained 100 VNN models on different permutations of the training set in OASIS-3, we use the mean of all VNN estimates as the VNN prediction for an individual. This VNN prediction is further leveraged to form brain age estimates and Δ-Age metrics. Figure 12a displays the plot for VNN predictions versus chronological age (ground truth) for the complete HC group. The Pearson's correlation between VNN prediction and chronological age (ground truth) for HC group was 0.486, which was similar to that reported in Section 4.1. VNN outputs clearly under-estimated the chronological age for older individuals and over-estimated the chronological age for individuals on the younger end of the age distribution for HC group.

Figure 12b displays the plot for VNN predictions versus chronological age (ground truth) for the complete AD+ group. The Pearson's correlation between VNN prediction and chronological age (ground truth) for AD+ group was 0.28. We further note that the VNN architecture and our analysis of regional residuals helped quantify the contribution of each brain region to a data point in Fig. 12a and Fig. 12b. Hence, the scatter plot in Fig. 12b could be affected by larger contributions of certain brain regions for AD+ group relative to the HC group.

Figure 12c illustrates the box plots of residuals evaluated by the difference between VNN predictions and chronological age for HC and AD+ groups. Figure 12c suggests that the chronological age

for AD+ group was underestimated as compared to that for HC group. This observation was also expected since AD+ group is significantly older than the HC group. However, we expect that the robust elevated regional residuals from brain regions in Fig. 2b mitigated the under-estimation effect due to higher age of AD+ group to some extent.

Figures 12d-f display the results after age-bias correction is applied to the VNN outputs. As expected, the brain age for HC group in Fig. 12d is largely concentrated around the line of equality ($x = y$ line). In contrast, the brain age for AD+ group in Fig. 12e is concentrated above the line of equality. These effects manifest into the box plots for Δ-Age in Fig. 12f where we observe the AD+ group to have elevated Δ-Age as compared to HC group.

VNN architecture facilitated isolation of the effects of accelerated aging before age-bias correction was applied. Hence, the transformation of VNN outputs to brain age from Fig. 12a-c to Fig. 12d-f was not surprising. However, such insights may be infeasible for machine learning approaches that lack transparency and hence, the impact of deviations due to neurodegeneration from the healthy control population cannot be interpreted or isolated. In this context, if the learning model was a black box, Fig. 12a-c may appear to be counter-intuitive to the goal of detecting accelerated aging in the AD+ group and the effect of age-bias correction can be unclear, thus, leading to several criticisms [23].

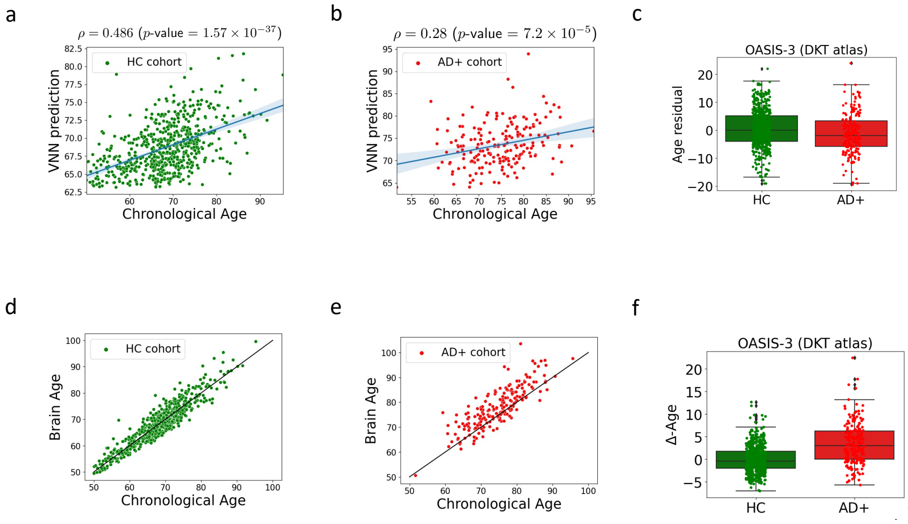

Figure 12: **Supplementary figures to Fig. 2.** Panel **a** displays the plot of VNN prediction versus chronological age for HC group. VNN predictions were obtained as the average of the outputs of 100 nominal VNNs that were trained on OASIS-3 and operated on the anatomical covariance matrix $\mathbf{C}_{\mathsf{HA}}$. Panel **b** displays the results similar to that in panel **a** for the AD+ group. The solid line in panels **a** and **b** is the least squares line. Panel **c** includes the boxplots for residuals derived from the difference between VNN predictions and chronological age for HC and AD+ groups. Panel **d** and **e** display the plots for brain age versus chronological age for HC and AD+ groups, respectively. The solid line in panels **d** and **e** is the identity line. Panel **f** displays the box plots for Δ-Age in HC and AD+ groups.

J Adaptive readouts may penalize the interpretability of regional residuals and Δ-Age

Thus far, we have focused on VNNs that operate with a non-adaptive readout (unweighted average) function. However, it is expected that the performance of the VNNs on their original task of chronological age prediction could be improved significantly with the help of an adaptive readout function. Our experiments showed that this was indeed the case. If a single-layer fully connected perceptron consisting of 10 neurons was added to the VNNs with the same architecture as the ones that were trained on OASIS-3 dataset, we could improve the performance on the chronological prediction task. For 100 VNNs with adaptive readout that were trained on random permutations of the training data, the median MAE for the HC group was 4.64 years, which was significantly smaller than the MAE achieved by VNNs with non-adaptive readouts (Section 4.1). Among the 100 VNN models with adaptive readouts, we analyzed the regional residuals for one VNN model with adaptive readout that had the best performance on chronological age prediction in HC group (test set: MAE = 4.17 years, Pearson's correlation between prediction and ground truth = 0.73; complete HC group: MAE = 4.26 years, Pearson's correlation between prediction and ground truth = 0.725). Our regional residuals revealed no significant difference between the regional residuals for AD+ group and HC group. This observation suggested that VNN lost its interpretability due to the addition of adaptive readout function. Moreover, we also observed a diminished gap between Δ-Age for AD+ and HC groups determined using this VNN model (Δ-Age for AD group: 1.58 ± 4.67 years, Δ-Age for HC group: 0 ± 3.45 years, Cohen's d = 0.384). The findings discussed here suggest that boosting the performance on chronological age prediction task by using an adaptive readout function may penalize the interpretability offered by VNNs with non-adaptive readouts and also diminish the Δ-Age gap between AD+ and HC groups.

K Results for randomly initialized VNNs

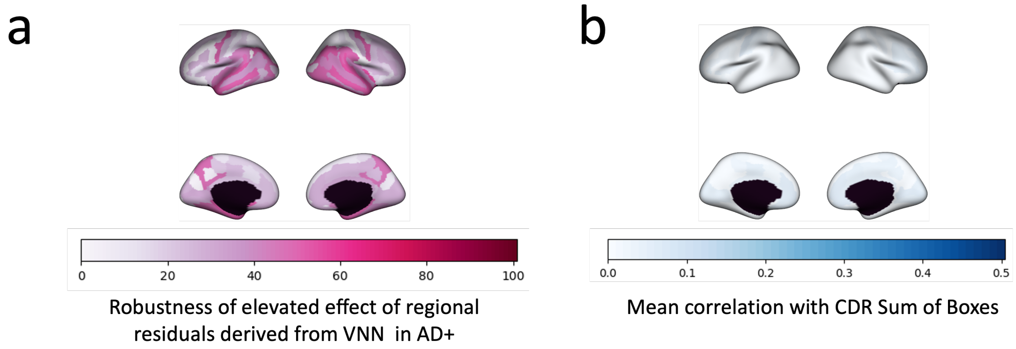

Figure 13: The results here were derived by VNN models that were randomly initialized and had the same architecture as those in Section 3.1.

L Regional profiles corresponding to elevated regional residuals in AD+ group are stable to the composition of data used to estimate anatomical covariance matrix $\mathbf{C}_{\mathsf{HA}}$

Recall that Δ-Age and associated regional profiles were evaluated using VNNs that operated upon a composite anatomical covariance matrix $\mathbf{C}_{\mathsf{HA}}$. We next checked whether the results derived from VNNs relevant to Δ-Age were stable to the changes in composition of the combined HC and AD+ groups used to estimate the anatomical covariance matrix. Note that the bilateral parahippocampal, entorhinal, subcallosal, and temporal pole regions are expected to be among the most relevant to Δ-Age in AD based on the results in Fig. 3.

We performed two sets of experiments. In the first set, we included the whole HC group and gradually varied the number of individuals from the AD+ group to be included to form the estimate $\mathbf{C}_{\mathsf{HA}}$. Figure 14a includes the results obtained from a randomly selected VNN model corresponding to the anatomical covariance matrix formed by different combinations of the individuals from HC and AD+ groups. The results in Fig. 14a display the brain regions whose regional residuals from AD+ group were higher than that in the HC group (Bonferroni corrected p-value < 0.05). The result obtained by the VNN when it used $\mathbf{C}_{\mathsf{HA}}$ estimated from all 611 HC individuals and 194 AD+ individuals forms the baseline to evaluate stability in this context. When the covariance matrix $\mathbf{C}_{\mathsf{HA}}$ was perturbed by using a smaller number of individuals from the AD+ group to estimate it, we observed that the significance of the relevant brain regions (parahippocampal, subcallosal and temporal pole) were preserved till exclusion of about 144 AD+ individuals from the estimate $\mathbf{C}_{\mathsf{HA}}$. Hence, the significant differences observed in the regional residuals for AD+ and HC groups in the aforementioned regions were robust to perturbations in $\mathbf{C}_{\mathsf{HA}}$ due to variability in the number of individuals from the AD+ group.

Figure 14b illustrates the results obtained for a similar experiment as above, with the difference that the regional residuals were evaluated for the VNN when the anatomical covariance matrix $\mathbf{C}_{\mathsf{HA}}$ was perturbed by reducing the number of individuals from the HC group used to estimate it. Using the result obtained for $\mathbf{C}_{\mathsf{HA}}$ estimated from 611 HC individuals and 194 AD+ individuals in Fig. 14a as the baseline, the results pertaining to ANOVA between regional residuals for AD+ and HC groups (with AD+ elevated as compared to HC) remained consistent as long as 100 or more individuals from HC group were included in forming $\mathbf{C}_{\mathsf{HA}}$. With less than 100 number of HC individuals included in $\mathbf{C}_{\mathsf{HA}}$, the results became noticeably less significant in precuneus and supramarginal regions in the left hemisphere.

In summary, the group differences observed between the regional residuals for AD+ and HC groups in OASIS-3 dataset were robust to perturbations in the covariance matrix $\mathbf{C}_{\mathsf{HA}}$ when it was perturbed from the baseline by using a different combination of HC and AD+ individuals to estimate it. However, we also remark that (nearly) complete exclusion of HC or AD+ groups from $\mathbf{C}_{\mathsf{HA}}$ resulted in loss of significance of the elevation in regional residuals in AD+ for various regions, including bilateral parahippocampal and temporal pole regions, and precuneus and supramarginal regions in the left hemisphere. Thus, both HC and AD+ groups were relevant to the anatomical covariance matrix $\mathbf{C}_{\mathsf{HA}}$ that resulted in regional profiles in Fig. 2a, and they were robust to the combination of individuals from HC and AD+ used to estimate $\mathbf{C}_{\mathsf{HA}}$.

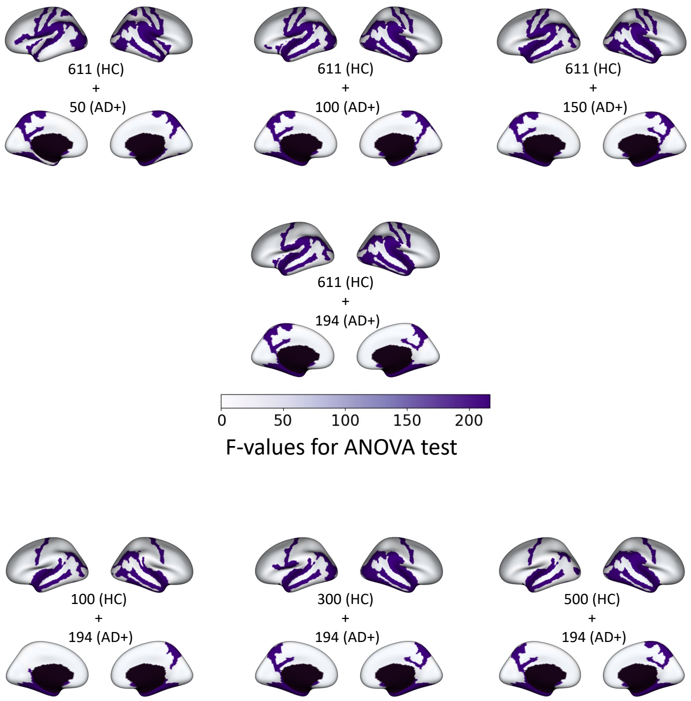

Figure 14: **Stability to perturbations in the anatomical covariance matrix for group differences between AD+ and HC groups observed in regional residuals.** For a VNN model that was trained to predict chronological age for HC group in OASIS-3 dataset, the regional residuals were first determined using the anatomical covariance matrix $\mathbf{C}_{\mathsf{HA}}$ formed by the cortical thickness data of complete OASIS-3 dataset (i.e., 611 HC individuals and 194 individuals in the AD+ group. The group differences in regional residuals between AD+ and HC group were investigated according to the procedure in subsection 3.2. In the procedure described therein, we evaluated the F-values for the ANOVA test between regional residuals for AD+ group and HC group. The brain regions associated with the regional residuals that were significantly elevated in AD+ group with respect to HC group are highlighted on the brain template. The stability of the group differences to perturbations in $\mathbf{C}_{\mathsf{HA}}$ was further investigated by varying the composition of cortical thickness data from AD+ and HC groups used to estimate $\mathbf{C}_{\mathsf{HA}}$. Figures in the top row display the results obtained via analysis of regional residuals by VNNs that processes the cortical thickness data from the complete OASIS-3 dataset over the anatomical covariance matrix $\mathbf{C}_{\mathsf{HA}}$ estimated from 611 HC individuals and a varying number of individuals from the AD+group. Figures in the bottom row illustrate the results of similar experiments, with the difference that the anatomical covariance matrix $\mathbf{C}_{\mathsf{HA}}$ was estimated using all 194 individuals in the AD+ group and varying number of individuals from the HC group. The results corresponding to $\mathbf{C}_{\mathsf{HA}}$ that was estimated using 194 AD+ individuals and 611 HC individuals formed the baseline for comparison for all scenarios.

M Δ-Age evaluation with anatomical covariance matrix from HC group

Using the anatomical covariance matrix derived only from the HC group (denoted by $\mathbf{C}_{\mathsf{H}}$) resulted in observations that were consistent with Fig. 2 with a slightly diminished difference in Δ-Age between HC and AD+ groups. Specifically, Δ-Age for AD+ group in this setting was 3.41 ± 4.57 years, which was significantly larger than that for the HC group (ANOVA: partial $\eta^2 = 0.141$, Cohen's $d = 0.88$). The correlation between Δ-Age and CDR sum of boxes scores in the AD+ group was 0.464. Thus, when the anatomical covariance matrix derived solely from the HC group was utilized in our brain age prediction framework, the magnitude of Δ-Age and its utility as a marker of dementia severity was slightly diminished as compared to the results in Fig. 2.

Figure 15a displays the regional profile associated with Δ-Age derived from VNNs with $\mathbf{C}_{\mathsf{H}}$ as the anatomical covariance matrix. Comparison with Fig. 2a reveals that the robustness of regional residuals being elevated in the AD+ group in subcallosal and temporal regions was preserved, but that in bilateral entorhinal and parahippocampal regions was diminished in this scenario. Figure 15b displays the distributions for Δ-Age in AD+ and HC groups.

The variation in the regional residuals associated with entorhinal and parahippocampal regions in Fig. 15a and Fig. 2a could be explained by investigating the eigenvectors of $\mathbf{C}_{\mathsf{H}}$. Specifically, Fig. 16a displays the associations between regional residuals for the AD+ group and the first 50 eigenvectors of $\mathbf{C}_{\mathsf{H}}$, where we observed that the third, second, and first eigenvectors of $\mathbf{C}_{\mathsf{H}}$ had the top three largest associations. Figure 16b plots the projections of the first three eigenvectors of $\mathbf{C}_{\mathsf{H}}$ on a brain template. Note that the eigenvectors $\mathbf{v}_3$ and $\mathbf{v}_2$ have the largest weights associated with the subcallosal region in the right hemisphere, which is consistent with the relevant eigenvectors of $\mathbf{C}_{\mathsf{HA}}$ in Fig. 3. However, unlike the eigenvectors of $\mathbf{C}_{\mathsf{H}}$ in Fig. 16b, the eigenvectors of $\mathbf{C}_{\mathsf{HA}}$ in Fig. 3b are also characterized by comparatively larger weights in the entorhinal and parahippocampal regions. Since parapippocampal and entorhinal regions are well known to be associated with disease onset and cortical atrophy [77], the anatomical covariance matrix $\mathbf{C}_{\mathsf{HA}}$ may provide a more holistic perspective to Δ-Age in AD.

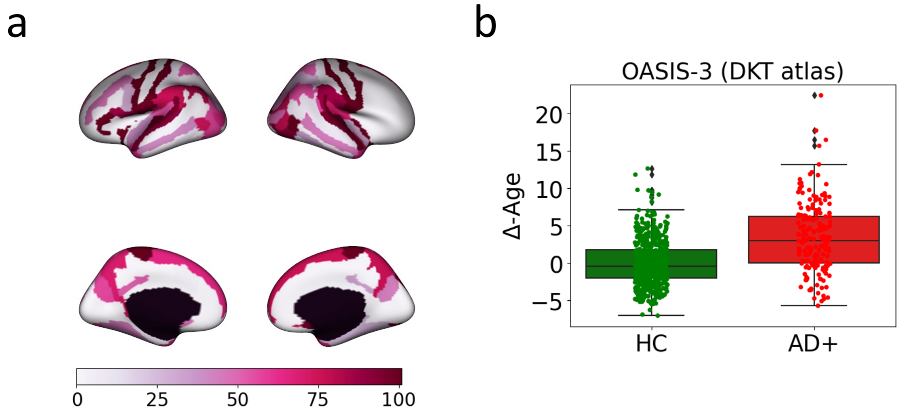

Figure 15: **Δ-Age results for anatomical covariance matrix from only HC group** ($\mathbf{C}_{\mathsf{H}}$)**.** Panel **a** projects the robustness of observing a significantly higher regional residual for AD+ group with respect to HC group for a brain region on the template. Panel **b** plots the Δ-Age distributions for AD+ and HC groups derived from VNNs with $\mathbf{C}_{\mathsf{H}}$ as the covariance matrix.

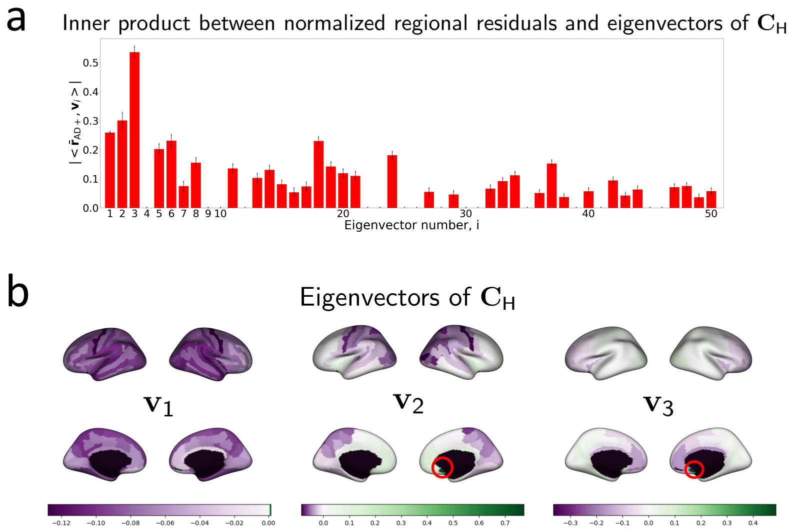

Figure 16: Panel **a** illustrates a bar plot for $|<\bar{\mathbf{r}}_{\mathsf{AD+}}, \mathbf{v}_i>|$ for $i \in \{1, \ldots, 30\}$, where $\mathbf{v}_i$ is the i-th eigenvector of covariance matrix $\mathbf{C}_{\mathsf{H}}$ associated with its i-largest eigenvalue. The bars are evaluated from the mean of $|<\bar{\mathbf{r}}_{\mathsf{AD+}}, \mathbf{v}_i>|$ obtained for individuals in the AD+ group (results for eigenvectors associated with coefficient of variation of $|<\bar{\mathbf{r}}_{\mathsf{AD+}}, \mathbf{v}_i>|$ larger than 30% excluded). For every individual in AD+ group, the association of its regional residuals with eigenvectors of $\mathbf{C}_{\mathsf{H}}$ were evaluated over 100 nominal VNN models (trained on the OASIS-3 dataset). The eigenvectors associated with top three largest values for $|<\bar{\mathbf{r}}_{\mathsf{AD+}}, \mathbf{v}_i>|$ are plotted on the brain template in Panel **b**. Subcallosal region in the right hemisphere was associated with the element with the largest magnitude in $\mathbf{v}_2$ and $\mathbf{v}_3$ and is highlighted with a red circle in the corresponding plots.
